# Tannins as Hemostasis Modulators

**DOI:** 10.3389/fphar.2021.806891

**Published:** 2022-01-13

**Authors:** Natalia Marcińczyk, Anna Gromotowicz-Popławska, Michał Tomczyk, Ewa Chabielska

**Affiliations:** ^1^ Department of Biopharmacy, Medical University of Białystok, Białystok, Poland; ^2^ Department of Pharmacognosy, Medical University of Białystok, Białystok, Poland

**Keywords:** tannins, platelets, thrombosis, coagulation, endothelium

## Abstract

The hemostasis system is often affected by complications associated with cardiovascular diseases, which results in thromboembolic events. Compounds of plant origin and plant extracts are considered as a promising source of substances that could modulate the functioning of the hemostasis system and thus reduce the risk of thromboembolism. Among them, tannins, which are plant-origin compounds with potential effects in hemostasis, deserve a special mention. This paper describes the hemostasis-modifying ability of three groups of tannins, namely ellagitannins, gallotannins, and procyanidins. The review highlights the desirable as well as undesirable influence of tannins on specific components of hemostasis, namely platelets, coagulation system, fibrinolysis system, and endothelium, and the multidirectional effect of these compounds on the thrombotic process. Studies performed under normal and pathological conditions such as diabetes or hypercoagulation are described, and the pathophysiology-dependent action of tannins is also highlighted. Most of the studies presented in the paper were performed *in vitro*, and due to the low bioavailability of tannins more studies should be conducted in the future to understand their actual activity *in vivo*.

## 1 Introduction

Hemostasis plays a vital role in ensuring the fluidity of blood in the intact vessel and preventing blood loss after its damage. The hemostatic balance is maintained by concurrent prothrombotic and antithrombotic reactions involving platelets, coagulation system, fibrinolysis system, and vessel wall. However, this balance shifts toward prothrombotic state in the event of inflammation, oxidative stress, and altered blood flow inside the vessel. Modifications in the functioning of the hemostasis system result in the development of serious and even fatal complications including myocardial infarction, deep vein thrombosis, and ischemic stroke which are associated with thromboembolism of affected vessels (Gale, 2011). The goal of antithrombotic therapy is to reduce the risk of thromboembolic events by lowering platelet activity, inhibiting the coagulation system, and improving endothelial function. However, due to adverse drug reactions or drug resistance, this aim is often not achieved (Mega and Simon, 2015). Therefore, there is a search for new substances of plant origin with beneficial effects in hemostasis. Tannins are a group of plant-origin substances that have the potential to prevent thromboembolic events. Tannins have been used throughout history for their ethnopharmacological properties in traditional medicine. Among the species from tannin-rich plants genus *Acacia*, *Agrimonia*, *Camellia*, *Geranium*, *Hamamelis*, *Krameria*, *Lythrum*, *Phyllanthus*, *Potentilla*, *Quercus*, *Rhus*, *Rubus*, *Sanguisorba*, or *Terminalia* are the most relevant from a medicinal point of view (Fraga-Corral et al., 2021). In general terms, these tannin-rich species have been described to treat gastrointestinal disorders or diseases (diarrhoea, congestion, anthelmintic), respiratory affections (pharyngitis, cold, expectorant and for sore throat), many skin issues such as eczema, ulcers, leukoderma, wounds), variable inflammatory processes (toothache, conjunctivitis, menstrual pain, haemorrhoids, smallpox, biliousness) or diabetes (Sieniawska, 2015). Our review discusses the influence of tannins on particular components of hemostasis, namely platelets, coagulation system, fibrinolysis system, and endothelium, under *in vitro* and *in vivo* conditions. Several studies have reported promising results observed with the application of tannins in animal models of thrombosis, which opens up a new perspective on plants as a source of antithrombotic compounds. The substances used in the presented studies were either extracted from plant materials or obtained by chemical synthesis based on plant-derived compounds.

## 2 Characteristics of Tannins

Tannins are polyphenolic compounds with a molecular weight ranging from 300 to 20,000 Da. Due to their high molecular weight, these substances are poorly absorbed from the gastrointestinal tract (Kiss and Piwowarski, 2019), where they are metabolized by the gut microbiota. It has been shown that the bioavailability of tannins is influenced by microbiota composition in the gut (Ou and Gu, 2014; Kiss and Piwowarski, 2019).

Based on their structure, tannins are classified into two groups: hydrolyzable and condensed. Hydrolyzable tannins are esters formed from glucose or other polyols and ellagic acid (ellagitannins) or gallic acid (gallotannins) (Khanbabaee and van Ree, 2001).

Ellagitannins are well soluble in water and undergo decay by acids and bases to release ellagic acid which is characterized with low solubility in water (Sójka et al., 2019). Moreover, they also form insoluble complexes with calcium and magnesium ions (Bock et al., 1981). Therefore, ellagitannins are poorly absorbed from the gastrointestinal (Serrano et al., 2016). The gut-derived metabolites of ellagitannins, called urolithins, have been extensively studied by researchers and reported to be responsible for the observed pharmacological effects *in vivo* (Espín et al., 2013). Urolithins are much better absorbed than parent compounds since their lower molecular weight and higher lipophilicity (Tomás-Barberán et al., 2017). Furthermore, increased consumption of ellagic acid does not improve its absorption but accelerates urolithins production (González-Sarrías et al., 2015). Urolithins are found in plasma, mainly in glucuronide conjugates (Piwowarski et al., 2017). Gallotannis are less soluble in water than ellagitannins (Sójka et al., 2019). Following oral administration, the high-molecular-weight gallotannins are enzymatically hydrolyzed by gut microbiota, leading to the formation of low-molecular-weight gallic acid, pyrogallol, and catechol which are found in plasma in glucuronidated, methylated, and sulfated forms (Scalbert et al., 2002; López de Felipe et al., 2014; Yang et al., 2020).

Condensed tannins or procyanidins are oligomers and polymers of flavan-3-ols, such as catechin, epicatechin, afzelechin, epiafzelechin, gallocetachin, and epigallocatechin (Aron and Kennedy, 2008). The core of flavan-3-ol is made of 15 carbon atoms arranged in three rings. The classification of procyanidins is based on the degree of polymerization of flavan-3-ol units and the type of bonds linking the units. Among the procyanidins, B-type compounds are dimer procyanidins which consist of two flavan-3-ol units linked by C4–C8 (procyanidins B1–B4) or C4–C6 (procyanidins B5–B8) interflavan bond. A-type procyanidins are also dimer procyanidins but they have an ether bond between carbons C2 and C7 in addition to C4–C8 interflavan bond. In turn, C-type procyanidins are trimer procyanidins which have only interflavan bonds (Fine, 2000; Aron and Kennedy, 2008). The presence of hydroxyl groups in the procyanidin monomers allows them to form oligomers with a high molecular weight (Xu et al., 2012). Procyanidins are slightly decomposed in the acidic environment of the stomach. Their dimers and trimers are absorbed in the small intestine, whereas the oligomers characterized by a higher degree of polymerization are not absorbed (Zhang et al., 2016). However, depolymerization of oligomeric procyanidins to monomers occurs to a negligible extent (Ou and Gu, 2014). A large part of the ingested procyanidins is metabolized by the colon microflora, resulting in low-molecular-weight compounds such as phenyl valerolactone, phenylacetic acid, and phenylpropionic acid (Zhang et al., 2016), but it is unclear whether these compounds are responsible for the effects of procyanidins observed *in vivo*. It was proposed that the activity of procyanidins may be related to their unknown microbial metabolites. Furthermore, the ability of procyanidins to alter the microbiota composition of the gut has been suggested to be responsible for their *in vivo* effects (Ou and Gu, 2014; Ma et al., 2020).

Studies on tannins’ pharmacokinetics are limited. Therefore, whenever it was possible, data on specific tannins or leading compound pharmacokinetics were included in this manuscript. Data Table 1 describes the chemical structure, bioavailability, metabolism, and metabolites of tannins.

**TABLE 1:** Structural characterization of tannins and their metabolism.

	Gallotannins	Ellagitannins	Procyanidins
Structure	Esters of gallic acid with monosaccharide (mostly beta-D-glucose)	Esters of ellagic acid with monosaccharide (mostly beta-D-glucose)	Oligomers and polymers of flavan-3-ols
			• B-type procyanidins: dimer procyanidins consist of two flavon-3-ol units with C4–C8 (procyanidins B1–B4) or C4–C6 (procyanidins B5–B8) interflavan bond
			• A-type procyanidins: dimer procyanidins with C4–C8 interflavan bond and an additional C2–C7 ether bond
			• C-type procyanidins: trimer procyanidins with interflavan bond
Bioavailability	• Low	• Low	• Degree of polymerization >3: not absorbed
Metabolism	• Hydrolysis in stomach to a lesser extent	• Metabolism by gut microbiota to urolithins	• Breakdown of procyanidins in the gastric environment
	• Mainly by gut microbiota		• Procyanidins are metabolized by colon microflora to low-molecular-weight compounds: phenyl valerolactone, phenylacetic acid, and phenylpropionic acid
Metabolites	• Low-weight gallotannins, pyrogallol, catechol, gallic acid: absorbed	• Urolithins well absorbed	• Dimers and trimers of procyanidin: absorbed to a small degree
			• Phenyl valerolactone, phenylacetic acid, and phenylpropionic acid: well absorbed

## 3 Tannins and Platelets

Platelets are anucleated cells derived from the cytoplasm of megakaryocytes. They play a key role in the primary hemostasis through the formation of the platelet plug at the site of vessel injury. In addition, platelets activate the coagulation factors on the phospholipids of their membranes and thus take part in the process of secondary hemostasis. Vessel injury results in the exposure of the components of the subendothelial matrix [e.g., collagen, tissue factor (TF), von Willebrand factor (vWf)] and activators present in the blood or released from platelets (e.g., thrombin, adenosine diphosphate (ADP), epinephrine). These in turn activate platelets through their specific receptors, leading to an increase in the intracellular calcium concentration followed by mobilization of phospholipids, release of arachidonic acid (AA), and secretion of dense granules (Harlan and Harker, 1981; Furie and Furie, 2007). Platelet activation is a complex process and involves the activation of multiple receptors and signaling pathways which are targets of antiplatelet drugs (Mega and Simon, 2015).

The metabolites of AA play an essential role in regulating the functions of platelets. During platelet activation, AA is released from the phospholipids of the cell membrane after their cleavage by phospholipase A_2_ (PLA_2_). The released AA is subsequently metabolized by cyclooxygenase (COX), which converts AA to eicosanoids [prostaglandins (PGs) and thromboxane A_2_ (TXA_2_)]. COX isoenzymes—COX-1 and COX-2—catalyze two different metabolic pathways: platelet-derived COX-1 generates TXA_2_, one of the most potent platelet activators, while COX-2 pathway leads to the production of proinflammatory PGs (Harlan and Harker, 1981). Due to the short half-life of TXA_2_, the synthesis of this eicosanoid is determined by measuring the concentration of its inactive, stable metabolite (TXB_2_) (Teng et al., 1997; Mattiello et al., 2009; Marcinczyk et al., 2017; Xie et al., 2017).

During activation, platelets change their shape from discoid to irregular form by cytoskeleton rearrangement. This contributes to the formation of stable platelet aggregates through secretion of granules and exposure of the membrane phospholipids which activate coagulation factors and thus accelerate fibrin formation (Hayashi et al., 2008). In platelet membrane, the phospholipids are asymmetrically distributed, with positive-charged and neutral phospholipids located in the outer leaflet of the membrane and negative-charged phospholipids such as phosphatidylserine (PS) in the inner leaflet. Sustained activation of platelets causes a disruption in the ordered arrangement of phospholipids. As a result, PS is irreversibly exposed from the inner leaflet to outer surface of the platelet membrane. Thus, PS is considered as a marker of irreversible activation of platelets (Hayashi et al., 2008). Table 2 summarizes the effect of tannins on platelet functions.

**TABLE 2:** Effect of tannins on platelet activity.

Tannin/extract/fraction	Dose/concentration	Effect		Material	References
Human studies *in vitro*					
bPGG from *Rhus verniciflua*	IC_50_ 1.6 × 10^−1^ mM	↓ aggregation induced by collagen (^a^0.2 μg/ml)		Whole blood	Jeon et al. (2006)
	IC_50_ 1.77 × 10^−1^ mM	↓ aggregation induced by ADP (15 µM)			
	IC_50_ 2.2 × 10^−1^ mM	↓ aggregation induced by AA (0.5 mM)			
	500 μg/ml	↓ P-selectin expression induced by ADP (10 µM)			
	500 μg/ml	↓ calcium mobilization		Platelet-rich plasma	
aPGG	1–10 µM	↓ expression of P-selectin induced by thrombin (0.1 U/ml)		Washed platelets	Perveen et al. (2011)
	0.3–10 µM	↓ secretion of ATP induced by thrombin (0.1 U/ml)			
	1–10 µM	↓ aggregation induced by ADP (5 µM)			
	0.3–10 µM	↓ aggregation induced by collagen (2.5 μg/ml)			
	10 µM	↓ decrease in cAMP level induced by thrombin (0.1 U/ml) and ADP (10 µM)			
		No changes in basal cAMP level in nontreated platelets			
	3–10 µM	↓ elevation of intracellular calcium concentration induced by thrombin (0.1 U/ml)			
	10 µM	↓ phosphorylation of Akt			
Pomegranate extract	3.3–6.6 µM polyphenol concentration	↓ aggregation induced by AA (two times the threshold concentration)		Washed platelets	Mattiello et al. (2009)
	2–6.6 µM polyphenol concentration	↓ aggregation induced by collagen (0.1–5 mg/L)			
	2–6.6 µM polyphenol concentration	↓ calcium mobilization induced by collagen (5 mg/L) or AA (0.05 mM)			
	1–2 µM polyphenol concentration	↓ TXA_2_ production in platelets treated with collagen (5 mg/L) or AA (0.05 mM)		Stirred washed platelets	
	2–6.6 µM polyphenol concentration	↓ H_2_O_2_ production in platelets stimulated with collagen (8 mg/L) or AA (0.05 mM)		Washed platelets	
Cinnamtannin 1B	10 µM	↓ thrombin (0.5 U/ml)-induced and H_2_O_2_ (10 µM)-induced activation and translocation of caspase 3 and caspase 9		Washed platelets	Bouaziz et al. (2007a)
	10 µM	↓ H_2_O_2_ (10 µM)-induced PS exposure			
	1–100 µM	↓ thrombin-induced (0.5 U/ml) PS exposure			
	0.1–10 µM	↓ thrombin (0.1 U/ml or 0.5 U/ml)-induced aggregation			Amor et al. (2007); Bouaziz et al. (2007b)
	10 µM	↓ ADP (10 µM)-induced aggregation			Amor et al. (2007)
		↓ thrombin (0.1 U/ml)-induced microtubular reorganization			Amor et al. (2007)
		↓ thrombin (0.1 U/ml)-induced Btk activation			Amor et al. (2007)
		↓ thrombin-induced (0.5 U/ml) ROS generation		Washed platelets from patients with type 2 diabetes	Bouaziz et al. (2007b)
		↓ thrombin-induced (0.5 U/ml) increase in intracellular calcium concentration			
		↓ thrombin (0.5 U/ml)-induced platelet aggregation			
Water–methanolic fraction of water–methanolic extract from *Medemia argun* nuts	5–50 μg/ml	↓ ONOO^−^ (100 µM)-induced protein nitration		Washed platelets	Morel et al. (2014)
		↓ protein carbonylation			
	0.5–50 μg/ml	↓ thiol group oxidation			
		↑ GSH level			
	5–50 μg/ml	↓ protein nitration		Plasma protein	
	0.5–50 μg/ml	↓ protein carbonylation			
		↓ thiol group oxidation			
		↑ GSH level			
Rugosin E from *Rosa rugosa*	Half-maximal effective concentration (EC50): 3.2 µM	Proaggregatory effect		Washed platelets	Teng et al. (1997)
Human study *in vivo*					
Cocoa flavanols and procyanidins	234 mg for 28 days	↓ ADP (8 µM)-induced aggregation		Whole blood	Murphy et al. (2003)
		↓ collagen (2 μg/ml)-induced aggregation			
		↓ collagen (2 μg/ml)-induced ATP release			
		↓ ADP (3 μM, 10 µM)-induced P-selectin expression			
		No changes in AA (1 mM)-induced aggregation			
Animal studies *in vitro*					
**Tannin/extract/fraction **	**Dose/concentration**	**Effect**	**Species/pathology**	**Material**	**References**
Methanolic fraction of aqueous extract from *Arbutus unedo* leaves	IC_50_ 0.7 g/L	↓ aggregation induced by thrombin (0.5 U/ml)	Rat	Washed platelets	Mekhfi et al. (2006)
bPGG from water–ethanolic extract from the roots of *Paeonia suffruticosa*	25–500 µM	↓ platelet adhesion to collagen (50 μg/ml)	Rat	Washed platelets	Qiu et al. (2017)
	100 µM	↑ expression of coronin 1B			
bPGG	400 µM	↓ PLCβ phosphorylation induced by thrombin (0.05 U/ml)	Rabbit	Washed platelets	Lee et al. (2014)
Rugosin E from *R. rugosa*	EC_50_ 1.5 µM	Proaggregatory effect	Rabbit	Washed platelets	Teng et al. (1997)
bPGG, O-trigalloyl-beta-D-glucose, isocorilagin isolated from the ethyl acetate extract from the seeds of *Picrorhiza kurroa*	All in 100 μg/ml	Inhibition of COX-1	COX-1 from ram seminal vesicles	*In vitro*	Zhang et al. (2004)
B-type procyanidin trimer and tetramer vs A-type procyanidins	IC_50_ 16 and 10 μM vs. 18–35% inhibition at 50 µM	Inhibition of PLA_2_	—	Fluorometric assay	Lambert et al. (2012)
Animal studies *in vivo*					
Extract from *Potentilla erecta* rhizome	400 mg for 14 days	↓ TXA_2_ synthesis	Rat	Whole blood	Marcinczyk et al. (2017)
		No changes in collagen (5 μL/ml)-induced aggregation			
	100–400 mg/kg for 14 days	↓ procoagulant platelet activity	STZ-induced diabetic rat	Whole blood	Marcinczyk et al. (2021a)
aPGG	Single oral administration at 20 mg/kg	↓ aggregation induced by ADP (1–10 µM)	Mouse	Platelet-rich plasma	Perveen et al. (2011)
		↓ aggregation induced by collagen (1–4 μg/ml)			
PGG isolated from the ethyl acetate fraction of water–ethanolic extract from *Paeoniae rubra* radix	7-day intravenous injection at 5 mg/kg	↓ TXB_2_ level	Rat injected with adrenaline	Serum	Xie et al. (2017)

a The concentration of agonist is mentioned in brackets.

### 3.1 Antiplatelet Activity of Tannins: *In Vitro* and *In Silico* Studies

#### 3.1.1 1,2,3,4,6-Pentagalloylglucose (PGG, Gallotannin)

After oral administration to rats the blood concentrations of PGG and its glucuronide were under limit of detection which suggested its low bioavailability. Incubation with rat fecal lysates led to decomposition of PGG to gallic acid and methyl gallate indicating the participation of gut microbiota in PGG metabolism. Both gallic acid and methyl gallate are characterized with higher bioavailability than PGG (Jiamboonsri et al., 2015). The antiplatelet activity of gallic acid (Chang et al., 2012) and methyl gallate (Chen et al., 2007) has been demonstrated *in vitro* which show their direct effect on platelets. However, due to the growing number of evidence for the influence of gut microbiota in biological active metabolites formation from plant-derived substances it cannot be excluded that some unknown metabolites of PGG are responsible for their activity *in vivo*.

PGG is the most studied tannin in the context of platelet activity. It occurs in two forms: beta pentagalloyl glucose (bPGG) and alpha pentagalloyl glucose (aPGG). bPGG is more prevalent in plants compared to aPGG (Cao et al., 2014). In the presented studies, the form of PGG used in the experiment was not specified by the authors, and so the exact form of PGG is not mentioned in this review. It has been shown that the application of bPGG obtained from the water–ethanolic extract of *Paeonia suffruticosa* Andrews (Paeoniaceae) roots inhibited the adhesion of rat platelets to collagen (Qiu et al., 2017). The inhibition of collagen-dependent platelet activation was also confirmed in an experiment which showed that the antiaggregatory effect of PGG isolated from the ethyl acetate fraction of water–methanolic extract from *Rhus verniciflua* Stokes (syn.: *Toxicodendron vernicifluum* Stokes, Anacardiaceae) bark on collagen-induced human platelet aggregation was 18-fold greater than the antiplatelet effect of acetylsalicylic acid ASA (500 μg/ml). PGG obtained from *R. verniciflua* showed higher inhibitory potential against ADP-induced and collagen-induced aggregation of human platelets than AA-induced platelet aggregation (Jeon et al., 2006). aPGG also inhibited ADP-induced as well as collagen-induced platelet aggregation, thrombin-induced secretion of P-selectin, and secretion of adenosine triphosphate (ATP) from dense granules of human platelets (Perveen et al., 2011). Furthermore, PGG from *R. verniciflua* reduced calcium mobilization and expression of P-selectin in human platelets previously activated by ADP (Jeon et al., 2006). In rabbit platelets, bPGG inhibited thrombin-induced phosphorylation and subsequent activation of phospholipase Cβ (PLCβ), leading to the secretion of dense granules (Lee et al., 2014; Flaumenhaft, 2017). It has also been shown that aPGG bound to the insulin receptor on the surface of human platelets and caused a reduction in their activity similar to insulin (Perveen et al., 2011). Moreover, the study revealed that the insulin-mimicking effect of aPGG was accompanied by the action of cyclic adenosine 3′,5′-cyclic monophosphate (cAMP), an important regulator of platelet activity. Increased intracellular concentration of cAMP in platelets triggers signaling pathways causing platelet inhibition by alleviating the rise in intracellular calcium concentration, whereas a decrease in intracellular cAMP level augments the activity of platelets. The authors observed that aPGG promoted the phosphorylation of the insulin receptor, and similar to insulin, inhibited the decrease in cAMP in ADP-activated or thrombin-activated platelets. Furthermore, aPGG interfered with collagen-stimulated Akt kinase phosphorylation as insulin did (Perveen et al., 2011). The activation of glycoprotein VI (GPVI), which is the main collagen receptor of platelets, triggers the activation of phosphatidylinositol 3-kinase (PI3k) and its receptor kinase (Akt kinase) pathway (Woulfe, 2010). PI3k-Akt signaling pathway is critical for the activation of platelet integrins which facilitate interactions between platelets, fibrin, and subendothelial matrix (Guidetti et al., 2015). It can be presumed that aPGG modulated PI3k-Akt signaling pathway in platelets and reduced the formation of stable platelet aggregates, thereby inhibiting Akt phosphorylation. Moreover, bPGG increased the expression of coronin 1B in rat platelets, which can be linked to hemostasis since rat platelets with elevated coronin 1B exhibited less adhesiveness to collagen (Ma et al., 2011; Qiu et al., 2017).

#### 3.1.2 Inhibition of PLA_2_

The influence of procyanidins on the AA cascade was investigated, with a focus on their structure. The inhibitory effect of procyanidins on PLA_2_ was analyzed using dye-labeled phospholipids as PLA_2_ substrates. It was found that oligomeric A-type procyanidins (parameritannin A1 and aesculitannin B1) were less potent in inhibiting PLA_2_ than oligomeric B-type procyanidins (procyanidin trimers and tetramers). In addition, the degree of inhibition within the group of procyanidins was dependent on the degree of their polymerization. Parameritannin A1 (tetramer) was identified to be a more potent PLA_2_ inhibitor than aesculitannin B1 (trimer), while B-type procyanidin tetramer was more potent than B-type procyanidin trimer. Furthermore, an *in silico* modeling study performed using a crystal structure of bee venom PLA_2_ revealed that oligomeric B-type procyanidins bound to the active site of PLA_2_ *via* Van der Waals interaction and sterically blocked the active site of the enzyme without modifying its structure. On the other hand, oligomeric A-type procyanidins altered the active-site structure of PLA_2_, causing a decrease in the binding affinity of the enzyme to its substrate as well as to oligomeric A-type procyanidins (Lambert et al., 2012).

#### 3.1.3 Inhibition of Arachidonic Acid Metabolism

The inhibitory effect of tannins on AA metabolism was demonstrated using an ethyl acetate extract obtained from the seeds of *Picrorhiza kurroa* Royle ex Benth. (Plantaginaceae). It was observed that bPGG, O-trigalloyl-beta-D-glucose (gallotannin), and isocorilagin (ellagitannin) inhibited the activity of COX-1 isolated from ram seminal vesicles. This effect was established by measuring O_2_ consumption during the transformation of AA to endoperoxide catalyzed by COX-1 (Zhang et al., 2004). In turn, the inhibitory effect of a pomegranate (*Punica granatum* L., Lythraceae) extract on AA metabolism was confirmed by decreased TXB_2_ concentration in the supernatant of stirred washed human platelets that had been preincubated with collagen or AA (Mattiello et al., 2009). A pomegranate extract rich in ellagitannins was obtained from whole-fruit pomegranate juice after its concentration and polyphenol recovery by absorption on resin with a water–ethanol solution (Aviram et al., 2008).

The main ellagitannin in pomegranate extract is punicalagin. However, after consumption of pomegranate extract, punicalagin was not detected in plasma and type of resulting metabolites differed among healthy individuals. Urolithin A, urolithin B, and their metabolites were found in the plasma. Some subjects did not produce urolithins whereas some subject had surprisingly high concentration of ellagic acid which is characterized with poor oral bioavailability (Mertens-Talcott et al., 2006). Based on the type of produced urolithins, humans can be stratified into several metabotypes that differ in the composition of the gut microbiota (Tomás-Barberán et al., 2014). Interindividual variability in pomegranate extract metabolism was probably due to the inclusion of different metabotypes in the study. It indicates that *in vitro* studies with ellagitannins does not provide information about their actual activity *in vivo*. Furthermore, because of the different metabotypes, the effect of ellagitannins after oral consumption may vary across individuals.

#### 3.1.4 Inhibition of Shape Change

Thrombin and hydrogen peroxide (H_2_O_2_) trigger similar molecular events as those associated with apoptosis. These include activation of apoptotic proteases called caspases and subsequent cytoskeleton depolymerization and PS exposure. In human platelets, caspase 3 and caspase 9 translocate to actin cytoskeleton where they catalyze the depolymerization reaction causing changes in the shape of platelets (Ben Amor et al., 2006). Cinnamtannin 1B (oligomeric A-type procyanidin trimer) isolated from *Laurus nobilis* L. (Lauraceae) wood extract, which was obtained using dichloromethane and ethyl acetate, decreased thrombin-induced and H_2_O_2_-induced irreversible human platelet activation, determined based on the degree of PS exposure. Cinnamtannin 1B decreased thrombin-induced and H_2_O_2_-induced activation of caspase 3 and caspase 9 and their translocation (as well as that of nonactive procaspases) to the cytoskeleton (Bouaziz et al., 2007a). Furthermore, it was reported that cinnamtannin inhibited the cytoskeleton reorganization of human platelets *in vitro* through the inhibition of Burton’s tyrosine kinase (Btk). This indicated that the antiplatelet activity of cinnamtannin 1B is related to its antioxidant properties due to the fact that the activation of Btk is a mechanism dependent on reactive oxygen species (ROS) (Amor et al., 2007). It has also been demonstrated that cinnamtannin B1 reduced ADP-induced and thrombin-induced aggregation of platelets obtained from healthy donors (Amor et al., 2007; Bouaziz et al., 2007b). In addition, cinnamtannin 1B was found to reverse enhanced calcium mobilization and thrombin-induced aggregation in platelets collected from patients with type 2 diabetes (Bouaziz et al., 2007b).

#### 3.1.5 Inhibition of Oxidative Stress

It has been shown that ellagitannin-rich water pomegranate extract decreased collagen-induced as well as AA-induced aggregation of human platelets and reduced their calcium mobilization. The antiplatelet effect of the pomegranate extract was associated with its antioxidant properties since the extract reduced H_2_O_2_ production in platelets (Mattiello et al., 2009). It is worth mentioning that agonists such as AA, thrombin, or collagen enhance the activity of platelets also by stimulating their ROS production (Pietraforte et al., 2014). This indicates that the antiplatelet effect of tannins is associated with their antioxidant properties and the resulting inhibition of ROS production in platelets. The inhibitory effect of tannins on oxidative stress also involves an increase in the concentration of antioxidant enzymes and prevention of protein modification resulting from stress. It has been shown that the water–methanolic procyanidin fraction of the water–methanolic extract obtained from *Medemia argun* (Mart.) Württemb. ex H.Wendl. (Arecaceae) nuts increased the level of the antioxidant glutathione (GSH) in peroxynitrate (ONOO^−^)-treated human platelets. Furthermore, the studied fraction alleviated ONOO^−^-evoked protein modifications, such as oxidation of thiol groups, nitration, and formation of carbonyl group, in the treated platelets (Morel et al., 2014).

### 3.2 Antiplatelet Activity of Tannins: *In Vivo* Studies

A study showed that consumption of cocoa flavanols and procyanidins for 28 days resulted in the attenuation of platelet activity in healthy subjects. The results indicated a decrease in collagen-induced and ADP-induced aggregation as well as a decrease in collagen-induced ATP secretion and ADP-induced expression of P-selectin without any changes in AA-induced aggregation. Consumption of cocoa flavanols resulted in the increased plasma concentration of epicatechin and catechin (Murphy et al., 2003) which display antiplatelet activity (Blache et al., 2002), and inhibit platelet-endothelium interaction (Carnevale et al., 2014).

Single oral administration of aPGG caused a reduction in ADP-induced and collagen-induced aggregation of mice platelets (Perveen et al., 2011). The inhibitory effect of tannins on AA metabolism has been confirmed *in vivo*. It was reported that after 14-day oral treatment with water–methanolic *Potentilla erecta* rhizome extract (*Potentilla erecta* (L.) Raeusch., Rosaceae), which was rich in ellagitannins and contained oligomeric procyanidins, a dose-dependent reduction in the concentration of TXB_2_ was observed in the supernatant of stirred rat whole blood (Marcinczyk et al., 2017). In the extract from streptozotocin (STZ)-induced diabetic rats, a decreased platelet procoagulant response was observed which was reflected as reduced PS exposure in the thrombus formed on collagen fibers. However, the antiplatelet effect decreased as the dose of the extract increased, indicating the opposite tendency to that seen in normoglycemia (Marcinczyk et al., 2021a).

PGG isolated from the ethyl acetate fraction of the water–ethanolic extract from *Paeonia rubra* radix (*Paeonia officinalis* subsp. *officinalis*, Paeoniaceae) decreased the level of TXB_2_ in the serum of rats with a procoagulant state (Xie et al., 2017). However, it should be mentioned that PGG was administered intravenously, and given the influence of gut microbiota in the formation of active tannin metabolites it is unclear whether omission of the gastrointestinal tract had an impact on the effect of PGG on platelets.

### 3.3 Proaggregatory Effect of Tannins

Rugosin E (ellagitannin) isolated from *Rosa rugosa* Thunb. (Rosaceae) enhanced the aggregation of rabbit and human platelets *in vitro*. It augmented TXB_2_ production in rabbit platelets but did not decrease the cAMP level or cause the cleavage of phosphoinositides, which are constituents of cell membrane phospholipids and play an important role in downstream signaling pathways leading to platelet activation (Teng et al., 1997).

In summary, the results on the inhibition of aggregation induced by various platelet agonists suggest that tannins could exhibit antiplatelet activity through different pathways. However, experiments analyzing agonists-induced aggregation use basic methods that allow determining whether the test substance has any effect on platelets. A few studies have evaluated in detail the mechanism underlying the action of tannins on platelets using molecular biology techniques. They indicate that the antiplatelet action of tannins is also associated with their antioxidant properties. Most of the presented studies were conducted on materials collected from humans, which might suggest the potential use of tannins in humans in the future. However, considering the low bioavailability of tannins, it is hard to determine the actual effect of these compounds on platelet activity since the majority of studies were carried out *in vitro* using nonmetabolized tannins. Moreover, it is not known whether the platelet-activating effect of rugosin E will be sustained under *in vivo* conditions or its action will shift toward antiplatelet effect.

It is also worth mentioning that tannins, along with other compounds, can inhibit the activity of platelets. A study showed that the methanolic fraction of an aqueous extract obtained from *Arbutus unedo* L. (Ericaceae) leaves inhibited thrombin-induced aggregation of rat platelets. This inhibitory effect was enhanced (from 65 to 75%) with the use of precipitated and methanol-dissolved tannins (Mekhfi et al., 2006).

Tannins have been found to form complexes with proteins, denaturate proteins (Hagerman, 1989), and interact with lipid bilayer (Yu et al., 2011). However, there is no data describing how these interaction affect platelet activity. The interactions between tannins with proteins can be both covalent and non-covalent, and occur through hydrophobic and electrostatic interactions or hydrogen bonds (Adamczyk et al., 2017). Tannins interact with proteins through the aromatic rings of the polyphenols and hydrophobic moieties of amino acids—mainly pyrrolidine rings of proline residues, while hydrogen bonds are formed between the hydroxyl groups and the acceptor site for hydrogen in a protein (Le Bourvellec and Renard, 2012). Hydrolysable tannins electrostatically bind to calcium dependent chloride channels located on the outer leaflet of the artificial cell membrane and inhibit them (Borisova et al., 2019). It could be one of the possible mechanism of the antiplatelet activity of tannins since inhibition of chloride channels in platelet leads to membrane depolarization and hinders agonist-induced hyperpolarization and PS exposure (Harper and Poole, 2013). Proline rich proteins are prone to form complexes with tannins (Lu and Bennick, 1998). Perhaps another mechanism of antiplatelet activity of tannins may be interaction with proline-rich tyrosine kinase 2 which is responsible for signal transduction during platelet activation (Ohmori et al., 2000).

The effect of tannins on the membrane fluidity seems to be very important in the mechanism of their action. Hydrophobic PGG can increase plasma membrane fluidity by penetration to hydrophobic part of artificial lipid bilayer whereas polar procyanidin trimer cannot (Yu et al., 2011). Data on the impact of plasma membrane fluidity in platelet activity are inconsistent. On the one hand, the antiplatelet drugs like ASA or ticagrelor rise the rigid of the platelets plasma membrane (Watała and Gwoździński, 1993; Lagoutte-Renosi et al., 2021) and platelets from type 1 diabetic patients have increased fluidity of the membrane (Mazzanti et al., 1997). On the other hand, platelet membrane is less fluid in patients who have hypertension, diabetes and also smoke (Nanetti et al., 2008). Therefore the changes in the platelet membrane fluidity caused by tannins in relation to platelet activity should be considered individually.

## 4 Tannins and Red Blood Cells

Red blood cells (RBC) play an important role in thrombus development. They increase the size of the thrombus, and during clot retraction, change their shape to “polyhedrocytes” making the thrombus more compact and more resistant to fibrinolysis. It has been shown that the phospholipids present on erythrocyte membrane act as catalytic surface for blood coagulation reactions *in vitro*. Furthermore, ADP released from erythrocytes and hemoglobin potentiate the activation of platelets (Litvinov and Weisel, 2017). In patients with type 2 diabetes, the shape of RBC is altered mainly by the reorganization of membrane phospholipids and deformation of membrane. PS translocates from the inner to the outer membrane leaflet resulting in erythrocyte death or eryptosis. An *in vitro* study demonstrated that oligomeric procyanidin extract obtained from *Pinus massoniana* Lamb. (Pinaceae) bark (9 μg/ml) improved the membrane elasticity of erythrocytes collected from patients with type 2 diabetes. The membrane of the treated erythrocytes was found more fluid and appeared smoother with a lesser degree of eryptotic changes in comparison to nontreated erythrocytes. The antioxidant mechanism of extract is liked to extensive presence of hydroxyl groups in oligomeric procyanidins (Visser et al., 2017). Due to the low bioavailability it is unlikely that intact oligomeric procyanidins would reach significant plasma concentration after oral administration.

## 5 Tannins and Coagulation System

The current model of coagulation, called cell-based coagulation model, implies that a complex of TF and factor VIIa (TF/VIIa) triggers blood coagulation reactions. TF is expressed on vascular smooth muscle cells, adventitial fibroblasts, many nonvascular cells, and microparticles circulating in the blood (Hoffman, 2001).

The former model of coagulation, called cascade model, divides the coagulation process into intrinsic pathway and extrinsic pathway which converge in a common pathway. Due to some clinical outcomes, this coagulation model is no longer applicable. However, the routine coagulation tests such as partial thromboplastin time (PT) and activated partial thromboplastin time (APTT) are based on the cascade model (Hoffman, 2001). APTT is affected by abnormalities in the intrinsic and common pathway of coagulation which involves changes in the levels of factors VIII, IX, XI, XII, and X and fibrinogen. This test is used to monitor unfractionated heparin therapy. PT is affected by changes in the levels of factors II, V, and VII and fibrinogen (Levy et al., 2014). Thrombin time (TT) is used to assess the time of conversion of fibrinogen to fibrin, and changes in its value indicate fibrin formation disorders and qualitative as well as quantitative fibrinogen abnormalities (Undas, 2017).

The cell-based and cascade models of coagulation eventually come down to the activation of factor X. The active form of factor X (Xa) converts prothrombin to thrombin, which subsequently transforms soluble fibrinogen into insoluble fibrin stabilizing thrombus (Hoffman, 2001).

### 5.1 Anticoagulant Activity of Tannins: *In Vitro* Studies and *In Silico* Studies

It was shown that bPGG (0.5 mM) isolated from a methanolic extract obtained from the whole plant of *Geum japonicum* Thunb. (Rosaceae) prolonged APTT, PT, and TT in rabbit plasma (Dong et al., 1998). Similarly, bPGG isolated from the ethyl acetate fraction of the water–methanolic extract from *Pelargonium inquinans* (L.) L’Hér. (Geraniaceae) leaves prolonged APTT and TT in human plasma. The anticoagulant effect of 1 μg of bPGG was reported to be comparable to that of 0.063 units of heparin for APTT and 2.73 units of heparin in the case of TT (Ji et al., 2005).

Ellagitannins isolated from the butanolic fraction of the methanolic extract obtained from the whole plant of *G. japonicum*, which included pedunculagin, tellimagrandin II, casuariin, and 5-desgalloylstachyurin (all at a concentration of 1 mM), prolonged PT, APTT, and TT in rabbit plasma. However, this effect was not as pronounced as the APTT- and TT-prolonging effect of heparin (1–500 μg/ml). Furthermore, the isolated ellagitannins reduced thrombin activity (half-maximal inhibitory concentration, IC_50_ 0.18, 0.07, 0.28, and 0.19 µM, respectively) and Xa activity (IC_50_ 0.56, 0.21, 0.72, and 0.28 µM, respectively). In addition, pedunculagin bound directly to the active site of thrombin in a competitive manner. Other compounds acted as mixed noncompetitive inhibitors of thrombin and inhibited fibrinogen hydrolysis catalyzed by thrombin (IC_50_ 0.014, 0.059, and 0.026 µM, respectively) more efficiently than pedunculagin (IC_50_ 0.15 µM, not significant). This effect was assessed using methods in which the activity of thrombin or factor Xa was measured from the absorbance of a chromogenic product resulting from thrombin-catalyzed or factor Xa-catalyzed reaction (Dong et al., 1998).

Among above mentioned ellagitannins pedunculagin was subjected to the incubation with human gut microbiota. It has been shown that dependent on the metabotype different urolithins were produced (Piwowarski et al., 2016). It means that different gut microbiota composition affect the production of metabolites responsible for biological activity. It is difficult to predict, whether pedunculagin will inhibit thrombin and Xa activities *in vivo* due to its low bioavailability and the possibility of formation of various metabolites.

Using a chromogenic substrate, it has been shown that the procyanidin fraction of an aqueous extract of *Brownea grandiceps* Jacq. (Fabaceae) flowers attenuated the activity of factor Xa (IC_50_ 237.08 μg/ml) (Pereira et al., 2017). Procyanidin B2 was also found to act as an inhibitor of factor Xa. *In silico* modeling indicated that procyanidin B2 blocked the substrate-binding site of Xa. This inhibitory effect of procyanidin B2 (IC_50_ 1.2 µM) was also confirmed using a chromogenic substrate for Xa (Bijak et al., 2014).

Based on the measurement of radioactivity, the bioavailability of [^14^C] procyanidin B2 in rats after oral administration was 82%. Estimation of radioactivity alone does not provide information whether the parent compound or metabolite is present in blood. Significant peaks of radioactivity were detected in blood 0.5 h and 5–6 h after oral administration. This, together with radioactivity intensity may suggest absorption of intact compound from the upper gastrointestinal tract and extensive absorption of low molecular weight metabolites and phenolic acids from colon (Stoupi et al., 2010). Therefore, it is likely that procyanidin B2 will inhibit Xa *in vivo* but activity of procyanidin B2 in association with its metabolites is unknown.

Although factor VII plays a crucial role in the cell-based coagulation model, the effect of tannins on its activity has not been analyzed in studies conducted so far. However, one study reported that hamamelitannin (ellagitannin) was capable of inhibiting factor VII. The active form of plasma hyaluronan-binding protein (PHBP) converts factor VII to VIIa. The PHPB autoactivates itself from the pro-PHBP form, and this process is accelerated by polyamines such as spermidine or heparin. One of the roles of heparin in the autoactivation of pro-PHBP is to determine the scaffold for the autoactivation reaction, whereas spermidine is mainly involved in intramolecular interaction within the pro-PHBP. It has been shown that hamamelitannin (IC_50_ 0.19 µM) inhibited spermidine-enhanced autoactivation of human PHBP but did not affect heparin-enhanced autoactivation. This indicates the selective action of hamamelitannin and its ability to indirectly modify the activity of factor VII (Yamamoto et al., 2011).

### 5.2 Anticoagulant Activity of Tannins: *In Vivo* Studies

bPGG has been shown to prolong APTT, PT, and TT and decrease the concentration of fibrinogen (after seven intravenous administrations, 5 mg/kg, 24-h interval) in plasma collected from rats that had been injected with adrenaline in order to obtain blood with a high coagulation status (Xie et al., 2017). These results can be considered as prospective and to indicate the therapeutic potential of bPGG since coagulation attenuation was observed in the model with enhanced coagulation system activity.

A study on apolipoprotein E-deficient mouse (Apoe^–^/^–^) model of atherosclerosis showed that 8-week standard diet supplementation with ellagitannin-rich walnuts (*Juglans regia* L., Juglandaceae, 1.2 g homogenized walnuts per 5 g diet) led to a moderate reduction in prothrombin level and increase in the antioxidative capacity in plasma. TF-induced thrombin generation test performed in platelet-free plasma indicated that in the group treated with walnuts-supplemented diet the thrombin generation potential was reduced. A slight decrease in thrombin generation did not cause any change in the levels of other coagulation-related parameters such as fibrinogen and factor V. Similarly, no changes were observed in platelet-related parameters such as GPIIb/IIIa activation, platelet adhesion to collagen, PS exposure and platelet α-granule secretion after activation with ADP, convulxin or PAR4 peptide (Nergiz-Ünal et al., 2013). Urolithin A, urolithin B, urolithin C, and urolithin D were found in healthy individuals’ plasma after walnuts consumption. However, the type of produced urolithin differed between subjects (Pfundstein et al., 2014). It suggests that urolithins could display anticoagulant activity but the relationship between type of produced urolithins and observed effects is not known.

### 5.3 Procoagulant Activity of Tannins *in Vivo* Studies

**FIGURE 1:**
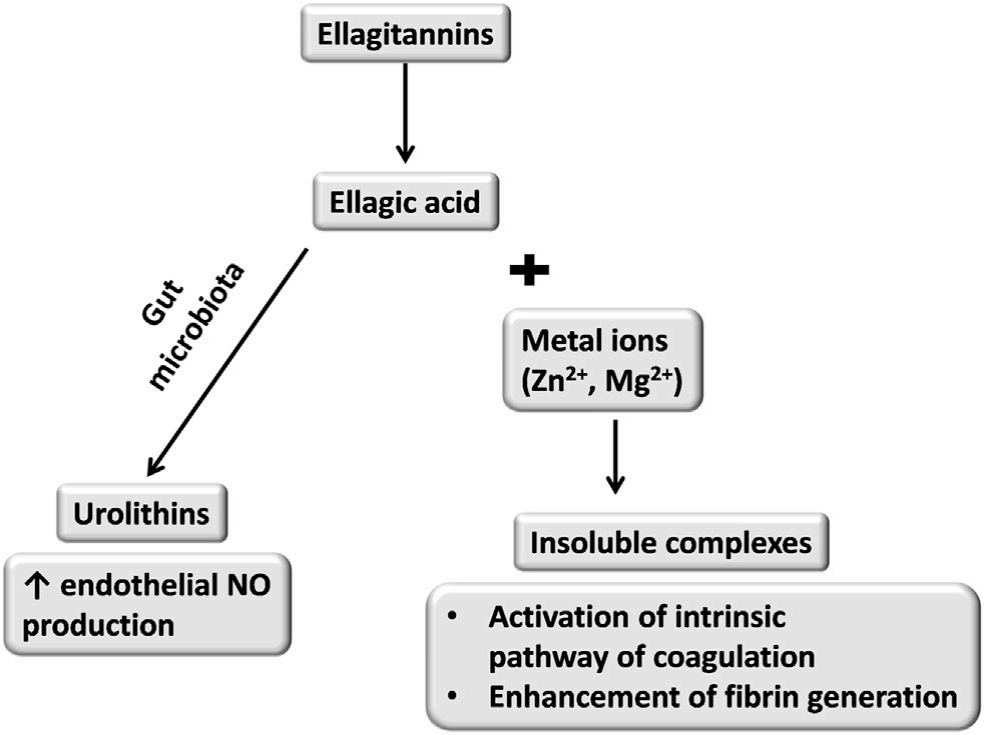
Possible effects of ellagitannins metabolism and resulted dual activity towards hemostasis (Bock et al., 1981; Spigoni et al., 2016).

Regardless of the antiplatelet effect, 14-day oral treatment (400 mg/kg) with *P. erecta* rhizome extract accelerated the rate of fibrin formation without any changes in PT and APTT. This effect was most likely to be associated with a slight increase in the intrinsic coagulation pathway which was not reflected by APTT (Marcinczyk et al., 2017). In STZ-induced diabetes in rats, administration of *P. erecta* rhizome extract accelerated fibrin formation in platelet-poor plasma (100, 200 mg/kg) and platelet-rich plasma (100 mg/kg, 200, 400 mg/kg) (Marcinczyk et al., 2021a). Ellagic acid released after the breakdown of ellagitannins forms insoluble complexes with zinc or magnesium ions. It has been shown that the complexes of ellagic acid with positive-charged ions accelerated the intrinsic coagulation pathway (Figure 1) (Bock et al., 1981).

It seems that a small amount of ellagic acid absorbed from the intestine formed insoluble complexes in plasma which enhanced the coagulation process, but this hypothesis needs to be verified.

In summary, the effects of tannins on coagulation were primarily assessed by measuring the routine coagulation parameters APTT, PT, and TT *in vitro*, while some *in vivo* studies have also been conducted. However, the inhibition of specific coagulation factors was analyzed only by *in vitro* and *in silico* experiments and the observed effects were not confirmed *in vivo*.

## 6 Tannins and Fibrinolysis System

The fibrinolysis system is responsible for dissolving a blood clot and thus avoiding vessel occlusion as well as preserving the vessel patency. Plasmin, which is the key component of the fibrinolysis system, dissolves the clot by degrading the fibrin net. Plasminogen (inactive form of plasmin) binds to the fibrin net and is activated by two activators: tissue plasminogen activator (t-PA), the main activator released from endothelium, and urokinase-type plasminogen activator (u-PA). The action of these two activators is inhibited by plasminogen activator inhibitor 1 (PAI-1) which is the major inhibitor of the fibrinolysis system (Gale, 2011).

### 6.1 Profibrinolytic Activity of Tannins

It has been shown that corilagin (ellagitannin, 7.5–60 µM) isolated from a methanolic extract obtained from the aerial parts of *Phyllanthus urinaria* L. (Phyllanthaceae) increased t-PA activity and decreased PAI-1 activity in plasma collected from healthy rats and also reduced the activity of PAI-1 released from thrombin-stimulated rat platelets *in vitro* (Shen et al., 2003). The bioavailability of corilagin after oral administration to rats was 10.7% (Zheng et al., 2016). Metabolomic profiling analysis showed that urolithin D, gallic acid, ellagic acid are present in plasma after oral administration of corilagin to rats (Yisimayili et al., 2019).

Oral administration of the extract from *P. erecta* rhizome for 14 days caused an increase in the activity of t-PA (200 mg/kg) in STZ-induced diabetic rats (Marcinczyk et al., 2021a).

### 6.2 Antifibrinolytic Activity of Tannins

Oral treatment with the extract from *P. erecta* rhizome for 14 days (400 mg/kg) resulted in a decrease in the activity of t-PA but no change in the concentration and activity of PAI-1 in plasma in normoglycemic rats (Marcinczyk et al., 2017). However, in STZ-induced diabetic rats, the extract inhibited clot dissolution which was reflected as prolonged euglobulin clot lysis time (200, 400 mg/kg) (Marcinczyk et al., 2021a).

Using a chromogenic substrate, it was established that the procyanidin fraction of the aqueous extract of *B. grandiceps* flowers inhibited the activity of plasmin (IC_50_ 47.80 μg/ml) and this effect was more pronounced than the inhibition of factor Xa (Pereira et al., 2017). Under *in vitro* condition, the studied fraction did not affect the activity of other fibrinolytic system components such as t-PA and u-PA. Brownplasmin was the main compound responsible for antifibrinolytic activity. It consists of flavan-3-ols of catechin linked with B-type bond which form heptadecamers (Pereira et al., 2017).

In summary, the effects of tannins on the fibrinolysis system have been investigated by a few studies. In addition, the number of reports describing the profibrinolytic and antifibrinolytic effects of tannins is similar. However, there are no data confirming the beneficial effect of tannins on the fibrinolytic system under pathological conditions. Therefore, it is important to investigate the activity of tannins in impaired fibrinolytic system and assess whether it is possible to achieve the beneficial effect of this compound under such conditions.

## 7 Tannins and Endothelium

Endothelium is a monolayer of cells lining the interior of the vessel wall. It plays a vital role in maintaining hemostasis and preventing its disruption associated with the release of antithrombotic factors such as nitric oxide (NO), prostacyclin (PGI_2_), and t-PA and the presence of ectonucleoside triphosphate diphosphohydrolase-1 (CD39) (Zhang, 2008; Antonioli et al., 2013). Inflammation or oxidative stress changes the phenotype of endothelium from antithrombotic to prothrombotic which is manifested by impaired synthesis of NO and PGI_2_, impaired platelet and leukocyte adhesion, disturbed fibrinolysis, and enhanced blood coagulation (Zhang, 2008).

### 7.1 Increased Production of NO and PGI_2_

Under physiological condition, endothelial nitric oxide synthase (eNOS) catalyzes the production of NO from L-arginine (Tousoulis et al., 2012). PGI_2_ is produced in the endothelium by subsequent conversion of AA to prostaglandin H_2_ (PGH_2_) by COX-1 and conversion of PGH_2_ by prostacyclin synthase. Both NO and PGI_2_ are endothelium-derived substances which play a key role in maintaining hemostasis due to their potent antiplatelet and vasorelaxing properties (Mitchell et al., 2008).

bPGG isolated from *P. rubra* radix (5 mg/kg, i.v., once a day for 7 days) increased the plasma level of eNOS as well as the plasma level of 6-keto prostaglandin F1α (6-keto-PGF1α; a stable metabolite of PGI_2_) in hypercoagulant rats (Xie et al., 2017). It has also been shown that consumption of procyanidin-rich chocolate (37 g chocolate, 4 mg/g of procyanidins) for 2 days resulted in an increase in the plasma level of 6-keto-PGF1α in healthy individuals (Schramm et al., 2001).

### 7.2 Antioxidant Activity

Enhanced degradation of NO by ROS contributes to decreased NO bioavailability and eNOS uncoupling. Thus, reducing oxidative stress is one of the goals of endothelial-targeting antithrombotic therapy (Zhang, 2008).

The molecular mechanisms underlying the antioxidant activity of tannins have been analyzed *in vitro* studies. One of the main ROS sources in the cardiovascular system is reduced nicotinamide adenine dinucleotide phosphate (NADPH) oxidase. This enzyme catalyzes the formation of superoxide anion (O_2_^−^) by one-electron reduction of oxygen and can produce a large amount of cytotoxic O_2_^−^ (Sun et al., 2016). It has been shown that three aqueous procyanidin fractions (50–1,000 ng/ml) from grape pomace, which differed in the degree of polymerization and percentage of galloylation, inhibited O_2_^−^ production in human umbilical vein endothelial cells (HUVECs) both at the intracellular and extracellular level by inhibiting NADPH oxidase. The effect of these three fractions (at 1,000 ng/ml) on reducing the extracellular O_2_^−^ concentration was comparable to that of superoxide dismutase (reduces O_2_^−^ to H_2_O_2_, 100 U/ml), apocynin (NADPH inhibitor, 100 µM), and diphenyliodinum (DPI; NADPH inhibitor, 60 µM). In turn, the effect of the fractions (at 500 ng/ml) on reducing the intracellular O_2_^−^ concentration was comparable to that of apocynin (100 µM), plumbagin (NADPH inhibitor, 5 µM), and also DPI (60 µM). Furthermore, it was determined that their inhibitory effect on NADPH oxidase and thus O_2_^−^ production was independent of the effect of O_2_^−^ scavenging. This result was reinforced by the fact that a higher concentration of procyanidins was required to scavenge O_2_^−^ (25,000–10,000 ng/ml) than to inhibit NADPH oxidase (10–100 ng/ml) (Álvarez et al., 2012).

NO rapidly reacts with O_2_^−^ and forms reactive and cytotoxic oxidant NOOO^−^which is implicated in the pathophysiology of cardiovascular diseases (Zou et al., 2004). It has been shown that endothelial cells exposed to 3-morpholinosydnonimine (SIN-1; a donor of NO) in the presence of procyanidins isolated from grape seeds (1–20 µM) exhibited less necrotic changes and a decreased degree of lipid peroxidation compared to those exposed to SIN-1 alone. Microscopic examination revealed that procyanidins formed a protective layer on the surface of endothelium and prevented lipid peroxidation on endothelial cell membrane, but did not prevent intracellular oxidation (Aldini et al., 2003). As mentioned, due to the high degree of polymerization, it is unlikely that the studied procyanidins would form a layer on endothelial cells *in vivo* after oral administration. Grape seeds procyanidins consist of dimers, trimers and polymerized oligomers of monomeric catechin units. After consumption of grape seeds procyanidins the procyanidin B1 was found in human blood (Sano et al., 2003). Furthermore, in study with rats the presence of procyanidin B1, procyanidin B2, epicatechin, and gallocatechin in plasma was confirmed after oral intake (Cheng et al., 2020). It is worth mentioning that plasma concentrations of these compounds were higher in rats with Alzheimer’s disease in comparison with healthy rats. It indicated changed pharmacokinetics of grape seeds procyanidins in pathological state (Cheng et al., 2020).

Oxidative stress plays an important role in the formation of atherosclerotic plaque. It causes low-density lipoprotein (LDL) to undergo oxidation and form oxidized LDL (ox-LDL). Endothelial damage arising from the accumulation ox-LDL triggers an inflammatory cascade which leads to adhesion, activation, and accumulation of monocytes, platelets, and smooth muscle cells (Poznyak et al., 2021). Furthermore, a study reported that ox-LDL contributes to endothelial dysfunction by increasing ROS production (Magwenzi et al., 2015). The results showed that the procyanidin fraction of a water–ethanolic extract from *Croton celtidifolius* Baill. (Euphorbiaceae) bark reduced ROS production in ox-LDL-stimulated HUVECs (1–10 μg/ml). This indicates that procyanidins have the potential to prevent endothelial dysfunction caused by atherosclerosis (Hort et al., 2012).

Tannins have also been shown to enhance NO production by activating ROS-dependent signaling pathways that exert a protective effect on endothelial cells. During oxidative stress, PI3k-Akt pathway is activated and promotes phosphorylation and subsequent activation of eNOS (Liang et al., 2017). It was found that 2,3-cis procyanidins (isolated from the ethyl acetate fraction of a methanolic extract from *Nelia meyeri* leaves, syn.: *Nelia pillansii* (N.E.Br.) Schwantes, Aizoaceae) and 2,3-trans procyanidins (isolated from the ethyl acetate fraction of a methanolic extract from *Salix* spp. Bark, Salicaceae) induced the activation of PI3k-Akt pathway by enhancing Akt phosphorylation in HUVECs (0.1–0.2 mg/ml) (Kaufeld et al., 2013; Kaufeld et al., 2014). Similarly, procyanidin C1 (50 µM) increased the release of NO in the rat artery endothelial cells and this effect was associated with the activation of PI3k-Akt pathway which was manifested by increased phosphorylation of Akt and eNOS (Byun, 2012). It is possible that increase in NO production would be observed *in vivo* since Procyanidin C1 is absorbed from the gastroinstestinal tract (Wang et al., 2018).

### 7.3 Anti-inflammatory Activity

Inflammation can contribute to the loss of antithrombotic function which is reflected by increased TF expression, abnormal functioning of the fibrinolysis system and coagulation system, and enhanced leukocyte adhesion.

#### 7.3.1 *In Vitro* Studies

The anti-inflammatory activity of tannins has been demonstrated primarily *in vitro* inflammation models such as HUVECs treated with tumor necrosis factor (TNFα) or with lipopolysaccharide (LPS). TNFα is a proinflammatory cytokine, and an increase in its level during cardiovascular and metabolic diseases leads to endothelial dysfunction mainly through oxidative stress and intensified inflammatory response. This cytokine induces the expression of vascular cell adhesion molecule 1 (VCAM-1) and intercellular cell adhesion molecule 1 (ICAM-1), which are the markers of endothelial cell activation. The main role of these adhesion molecules is to contribute to the attachment of leukocytes to endothelium and enhance vascular permeability (Zhang, 2008). LPS, an endotoxin present in the outer membrane of Gram-negative bacteria, increases the expression of VCAM-1 and ICAM-1 and enhances the platelet–endothelium interaction, which results in the intensification of the thrombotic process (Sawa et al., 2008; Marcinczyk et al., 2021b).

It was shown that bPGG (100 µM) isolated from *P. suffruticosa* root cortex decreased TNFα-induced expression of VCAM-1 and ICAM-1 in HUVECs (Kang et al., 2005). Similarly, the procyanidin fraction of an acetone–water extract obtained from *Ribes nigrum* L. (Grossulariaceae) leaves (10, 30, and 60 μg/ml) inhibited ICAM-1 expression in TNFα-stimulated LT2 endothelial cells (Garbacki et al., 2005). In another study, tannin-rich polyphenol fractions of acetone–water extracts obtained from *Rubus caesius* L. and *Rubus idaeus* L. (Rosaceae) leaves increased ICAM-1 expression in unstimulated HUVECs (both fractions at concentrations of 2.5–15 μg/ml) and TNFα-stimulated HUVECs (both fractions at concentrations of 5–15 μg/ml) (Dudzinska et al., 2014). In HUVECs pretreated with LPS, procyanidin B2 (2 µM) decreased inflammasome NLPR3 expression, caspase 1 activation, and interleukin 1β (proinflammatory cytokine) concentration. Inflammasome NLRP3 is a multiprotein complex composed of sensor molecule, adaptor protein (AP-1), and caspase 1. Its main role is to trigger inflammatory responses including the production of proinflammatory cytokines. The expression of NLRP3 is regulated by AP-1 which is activated by ROS, LDL, and hypoxia. However, it is unclear whether the inhibition of AP-1 is caused by the inhibition of ROS production or by another mechanism involved in this effect (Yang et al., 2014).

Chronic hyperglycemia is one of the causes of inflammatory changes observed in the endothelium. Under hyperglycemic conditions, the transcription factor nuclear factor KB (NFKB) regulates the expression of ICAM-1 and VCAM-1, and protein kinase C (PKC) indirectly contributes to the increase in vascular permeability, enhancement of oxidative stress, and impairment of eNOS function (van den Oever et al., 2010). A study showed that grape seed procyanidins decreased the expression of ICAM-1 and VCAM-1, and reduced the activity of PKC (at 20 μg/ml) and NFΚB (at 20 and 40 μg/ml) in glucose-treated HUVECs. However, the procyanidins did not reverse the suppression of NO production in the studied cells (Jiang et al., 2015).

#### 7.3.2 *In Vivo* Studies

It has been demonstrated that 24-week oral treatment with grape seed procyanidins (250, 500 mg/kg) reduced the expression of VCAM-1 and ICAM-1 in aortas from STZ-induced diabetic rats. Because of low bioavailability of oligomeric procyanidins and their well-absorbed gut microbiota metabolites it is possible that different compounds caused reduction of VCAM-1 and ICAM-1 expression *in vitro* and *in vivo*. Furthermore, it is worth mentioning that procyanidin treatment was started at the early stage of diabetes, which indicates the potential of grape seeds procyanidins in the prevention of vascular complications of diabetes (Jiang et al., 2015). However, it is unknown whether these procyanidins would alleviate endothelial dysfunction associated with long-term diabetes complications.

### 7.4 Endothelium-dependent Vasorelaxant Effect

Hemodynamic conditions are one of the factors determining platelet–vessel wall interactions and shear-stress-induced platelet activation (Sakariassen et al., 2015). Furthermore, a decrease in blood flow accelerates thrombus formation (Zakrzeska et al., 2015). The vast majority of studies on tannins have focused on the NO-dependent and endothelium-dependent vasorelaxant response ignoring the changes in hemostasis parameters. However, since NO plays an important role in maintaining hemostasis, these studies are worth mentioning. Most of the studies presented in this section concern procyanidins (Goto et al., 1996; Kim et al., 2000; Côrtes et al., 2002; DalBó et al., 2008; Magos et al., 2008; Campana et al., 2009; Matsui et al., 2009; Okudan et al., 2011; Byun, 2012; Furuuchi et al., 2012; Hort et al., 2012; Kaufeld et al., 2013; Byun et al., 2014; Kaufeld et al., 2014; Novakovic et al., 2017; Pinna et al., 2017), while a few concern gallotannins (Kang et al., 2005; Beretta et al., 2009).

It has been shown that under *in vitro* conditions tannins exerted vasorelaxant effect by increasing NO synthesis in healthy rats (Goto et al., 1996; Kim et al., 2000; Côrtes et al., 2002; Kang et al., 2005; DalBó et al., 2008; Campana et al., 2009; Matsui et al., 2009; Byun, 2012; Byun et al., 2014) and pigs (Kaufeld et al., 2013; Kaufeld et al., 2014). Some experiments revealed that the vasorelaxant effect of tannins involved the activation of calcium channels and potassium channels (Matsui et al., 2009; Byun, 2012; Byun et al., 2014; Kaufeld et al., 2014), but one study excluded calcium channel involvement in this effect (Kim et al., 2000). Studies have also pointed out the relationship between the antioxidant properties of tannins and their NO-dependent relaxation effect (Kaufeld et al., 2013; Kaufeld et al., 2014).

It should be emphasized that NO-dependent vasorelaxation was observed to be enhanced under pathological conditions. Improvement in NO-vasorelaxant response was demonstrated *in vitro* in vessels harvested from patients who had undergone artery bypass grafting (Novakovic et al., 2017), rabbits after ischemia–reperfusion injury (Beretta et al., 2009), and rats with hypertension (Magos et al., 2008; Furuuchi et al., 2012). Restoration of NO-dependent vasorelaxation was confirmed *ex vivo* in vessels harvested from diabetic rats (Okudan et al., 2011; Pinna et al., 2017) and mice with hypercholesterolemia (Hort et al., 2012). Similar to studies under normal condition, studies on pathological conditions indicated that the vasorelaxant effect was linked to the inhibition of oxidative stress (Beretta et al., 2009; Okudan et al., 2011), with two studies confirming the involvement of potassium channel activation in this effect (Beretta et al., 2009; Novakovic et al., 2017). Furthermore, it has been demonstrated that the vasorelaxant effect of tannins was influenced by PGI_2_ (Novakovic et al., 2017).

### 7.5 Endothelial Purinergic Pathway

CD39 and CD73 are membrane-bound ectoenzymes expressed, among others, on the surface of endothelial cells and monocytes. The enzyme CD39 catalyzes the conversion of ATP and ADP to adenosine monophosphate (AMP), whereas CD73 hydrolyzes AMP into adenosine (Ado). ADP acts as a potent platelet agonist, whereas Ado inhibits the activity of platelets (Antonioli et al., 2013).

Incubation of HUVECs with the tannin-rich polyphenol fraction of the water–acetone extract from *R. nigrum* leaves (2.5–15 µg GAE/ml (GAE refers to mg gallic acid equivalent per g of dry weight of extract)) caused an increase in the expression of CD39, but the studied fraction (10, 15 µg GAE/ml) reduced the activity of CD39. The decreased activity of CD39 and the role of monocyte CD39 in thrombosis attenuation *in vivo* could indicate that the activity of human platelets was not reduced after incubation with HUVECs that had been previously treated with the studied fraction (2.5–15 µg GAE/ml). It was also established that the fraction (2.5–15 µg) increased the phosphorylation of eNOS at serine 1177 and decreased the phosphorylation at threonine 495, which are sites responsible for the activation and inhibition of the enzyme, respectively. However, the expression of eNOS in HUVECs was found to be unchanged (Luzak et al., 2014). Similar to the polyphenol fraction of *R. nigrum*, the tannin-rich polyphenol fraction of the water–acetone extract from *R. caesius* (10, 15 μg/ml) and *R. idaeus* (10, 15 μg/ml) leaves increased CD39 expression in HUVECs and decreased its activity (both extracts at a concentration of 15 μg/ml) (Dudzinska et al., 2014).

In summary, the effect of tannins on endothelial cells translates into an improvement in the production of NO and PGI_2_. The studies cited in the section explain the molecular mechanism underlying the antioxidant and anti-inflammatory activity of tannins. In addition, the vasorelaxant effect of tannins and the related mechanism are described. It should be noted that a few of the presented studies were carried out under pathological conditions which points out the possible therapeutic benefits of tannins. However, most of them were performed *in vitro* and further studies are therefore needed to establish the actual activity of tannins *in vivo*.

## 8 Tannins and Experimental Models of Thrombosis

Research on new substances that may have potential therapeutic effects on the hemostasis system primarily aimed at reducing the risk of cardiovascular events. Thus, studies performed on experimental thrombosis models are of particular value because they demonstrate the overall *in vivo* effect of the tested compounds on the thrombotic process which involves the activity of specific hemostasis elements. This review distinguishes two main types of thrombosis: venous thrombosis and arterial thrombosis. The section “Other models of thrombosis” describes models in which the mechanism of thrombus formation does not correspond with the pathophysiological mechanism of venous or arterial thrombosis. Furthermore, the models in which tannins were administered before thrombus induction are specified as preventive, while the models in which tannins were administered after thrombus formation are specified as therapeutic.

Table 3 lists the studies focusing on the activity of tannins in animal thrombosis models.

**TABLE 3:** Effect of tannins on experimental models of thrombosis.

Venous thrombosis				
Treatment	Model of thrombosis	Main outcome	Proposed mechanism	References
• Grape seeds procyanidins	• Venous thrombosis induced by *inferior vena cava* ligation in rats before treatment with grape seed procyanidins	• Decreased thrombus weight	• Improvement of endothelial function	Zhang et al. (2011)
• 14-Day oral treatment at a dose of 400 mg/kg after *vena cava* ligation	• Therapeutic model	• Suppression of P-selectin, vWf, VCAM-1, and ICAM-1 expression on vessel wall	• Anti-inflammatory effect	
		• Increased expression of ADAMTS13 (responsible for degradation of vWf multimers to shorter fragments, limits the procoagulant activity of vWf) on the vessel wall		
		• Increased endothelial cell integrity		
		• Counteraction against leukocyte adhesion to endothelium		
		• Decreased serum level of proinflammatory cytokines: IL-6, IL-8, and TNFα		
• Grape seed procyanidins	• Venous thrombosis induced by *inferior vena cava* ligation in rats after treatment with grape seed procyanidins	• No effect on thrombus weight	• Improvement of endothelial function caused mainly by the increase in NO release	De Curtis et al. (2003)
• 30-Day oral treatment at a dose of 3.4 g/rat or single intravenous injection at a dose of 5–15 mg/kg before *vena cava* ligation	• Preventive model	• Prolongation of bleeding time		
		• No changes in APTT, PT, and platelet adhesion to collagen		
Arterial thrombosis				
• Extract from *Potentilla erecta* rhizome	• Electrical-induced thrombosis, rat carotid artery	• Decreased thrombus weight	• Platelet inhibition	Marcinczyk et al. (2017)
• 14-Day oral treatment at a dose of 400 mg/kg	• Preventive model	• Prolonged time to artery occlusion	• Inhibition of thromboxane synthesis	
• Extract from *Potentilla erecta* rhizome	• Electrical-induced thrombosis, rat carotid artery	• Increased thrombus weight	• Enhanced fibrin formation	Marcinczyk et al. (2021a)
• 14-Day oral treatment at a dose of 200 mg/kg (STZ-induced diabetic rats)	• Preventive model		• Attenuated fibrinolysis	
• Corilagin	• Electrical-induced thrombosis, rat carotid artery. Therapeutic model	• Restoring the blood flow in the occluded rat carotid artery	• Enhancement of fibrinolytic response	Shen et al. (2003)
• Single intravenous injection at a dose of 1.25–5 mg/kg after thrombus formation		• Thrombolytic effect at a dose of 5 mg/kg was comparable to the thrombolytic effect after urokinase (20,000 U/kg) administration		
		• No changes in ADP-induced, AA-induced, or PAF-induced rabbit platelet aggregation *in vitro*		
• Grape seed procyanidins	• Helium-neon laser + Evans blue-induced thrombosis in a mouse carotid artery	• Decreased thrombus size	• Platelet inhibition	Sano et al. (2005)
• Single intraarterial injection at a dose of 20 mg/kg or two oral administrations (2 × 200 mg/kg with 30-min interval)	• Preventive model	• Reduced shear-stress-induced platelet activation *ex vivo* after repeated oral administration		
Other models				
• Extract from *P. erecta* rhizome	• Ferric chloride-induced thrombosis, mouse mesentery vein	• Decreased PS exposure	• Platelet inhibition	Marcinczyk et al. (2017)
• 2-day oral treatment at a dose of 400 mg/kg	• Parietal thrombus	• Reduced the extent of PS exposure at a dose of 400 mg/kg to the extent comparable to aspirin (one single oral administration, 100 mg/kg)	• Inhibition of thromboxane synthesis
	• Preventive model		
• Extract from *P. erecta* rhizome	• Laser-induced thrombosis	• Decreased thrombus area (100 mg/kg)	• Platelet inhibition	Marcinczyk et al. (2021a)
• 14-day oral treatment in STZ-induced diabetic mice	• Preventive model	• Decreased P-selectin secretion (100 mg/kg, 400 mg/kg)		

### 8.1 Venous Thrombosis

Deep vein thrombosis results from factors such as blood flow congestion, endothelial cell activation, and enhanced activation of the coagulation system. Reduced blood flow (achieved under experimental condition by venous ligation), hypoxia, or production of inflammatory mediators such as TNFα leads to the activation of endothelial cells, which is manifested by the expression of P-selectin, vWf, and TF on endothelial cells. P-selectin binds to its receptor (PSGL-1) expressed on the surface on leukocytes and contributes to the adhesion of leukocytes to endothelium and their subsequent recruitment into the growing thrombus. Increased expression of TF triggers fibrin formation and the incorporation of RBC into thrombus (Stone et al., 2017).

In a study on the rat model of deep vein thrombosis, ethanolic procyanidin extract obtained from grape seeds caused a reduction in venous thrombosis and endothelial cell activation and alleviated inflammation. *Vena cava* ligation was performed 14 days prior to the administration of procyanidin extract, which might indicate that procyanidins alleviated the prothrombotic shift of the endothelium as reflected by the decreased thrombus weight (Zhang et al., 2011).

In another study, grape seed procyanidins (type of extraction is not known) did not cause any change in the venous thrombus weight. Thrombosis was induced after 30 days of extract administration in healthy animals, and an increased NO concentration was observed without any change in venous thrombosis (De Curtis et al., 2003). This suggests that grape seed procyanidins could exhibit favorable effects under both normal and pathological conditions which indicate their potential preventive and therapeutic applications.

### 8.2 Arterial Thrombosis

In arterial thrombosis, platelets bind to subendothelial matrix at the site of vascular injury and form thrombus. Arterial thrombus is primarily composed of platelets and a minor proportion of erythrocytes. In an electrical-induced thrombosis model, electrical stimulation leads to vessel wall damage and subsequently thrombus formation (Marcinczyk et al., 2017). Using this model, a study demonstrated the thrombus-preventive properties of *P. erecta* rhizome extract in healthy rats (Marcinczyk et al., 2017). However, in the study on STZ-induced diabetic rats, this extract increased thrombus weight due to enhanced fibrin formation and attenuated fibrinolysis (Marcinczyk et al., 2021a). The findings of these studies indicate that the overall effect of the studied extract on thrombosis might vary depending on the pathological condition. Contrary to the *P. erecta* extract, corilagin injected after thrombus formation exhibited thrombolytic effect in rats which indicates its therapeutic potential (Shen et al., 2003).

The antithrombotic effect of grape seed procyanidins was demonstrated using a model of laser-induced thrombosis. In this model, Evans blue dye injected before laser irradiation absorbs the laser energy and converts it to thermal energy. Vessel damage occurs at the site of laser irradiation, and thrombus forms on the exposed subendothelial matrix (Sano et al., 2005).

### 8.3 Other Model of Thrombosis

The extract from *P. erecta* rhizome (400 mg/kg) decreased FeCl_3_-induced thrombosis in mice (Marcinczyk et al., 2017). However, in the study, thrombosis was induced in mesentery vein. In this thrombosis model, negative-charged erythrocytes are attached to the vessel wall by positive-charged Fe^3+^ ions which promote further adhesion of platelets and their activation. Coagulation reactions take place on exposed phospholipids (Ciciliano et al., 2015). The resulting parietal thrombus did not close the lumen of the vessel which allowed observing the variability in platelet activity within the thrombus and assessing the extent of irreversible platelet activation. In STZ-diabetic mice, the extract (100 mg/kg) decreased the area of laser-induced thrombus in the mesentery vein. The model used in the study was composed of platelets that did not undergo irreversible activation and reflected rapid platelet response to the vessel wall injury. Moreover, at a concentration of 400 mg/kg, the extract did not affect thrombus which indicates the different mechanisms of action of the extract in normoglycemia and diabetes. Furthermore, using this model, it was shown that the extract decreased the secretion of P-selectin (Marcinczyk et al., 2021a).

In summary, the antithrombotic effect of tannins can be attributed to platelet inhibition, endothelial function improvement, or increased activity of the fibrinolysis system. The prothrombotic effect of tannins has also been reported.

## 9 Conclusion

**FIGURE 2:**
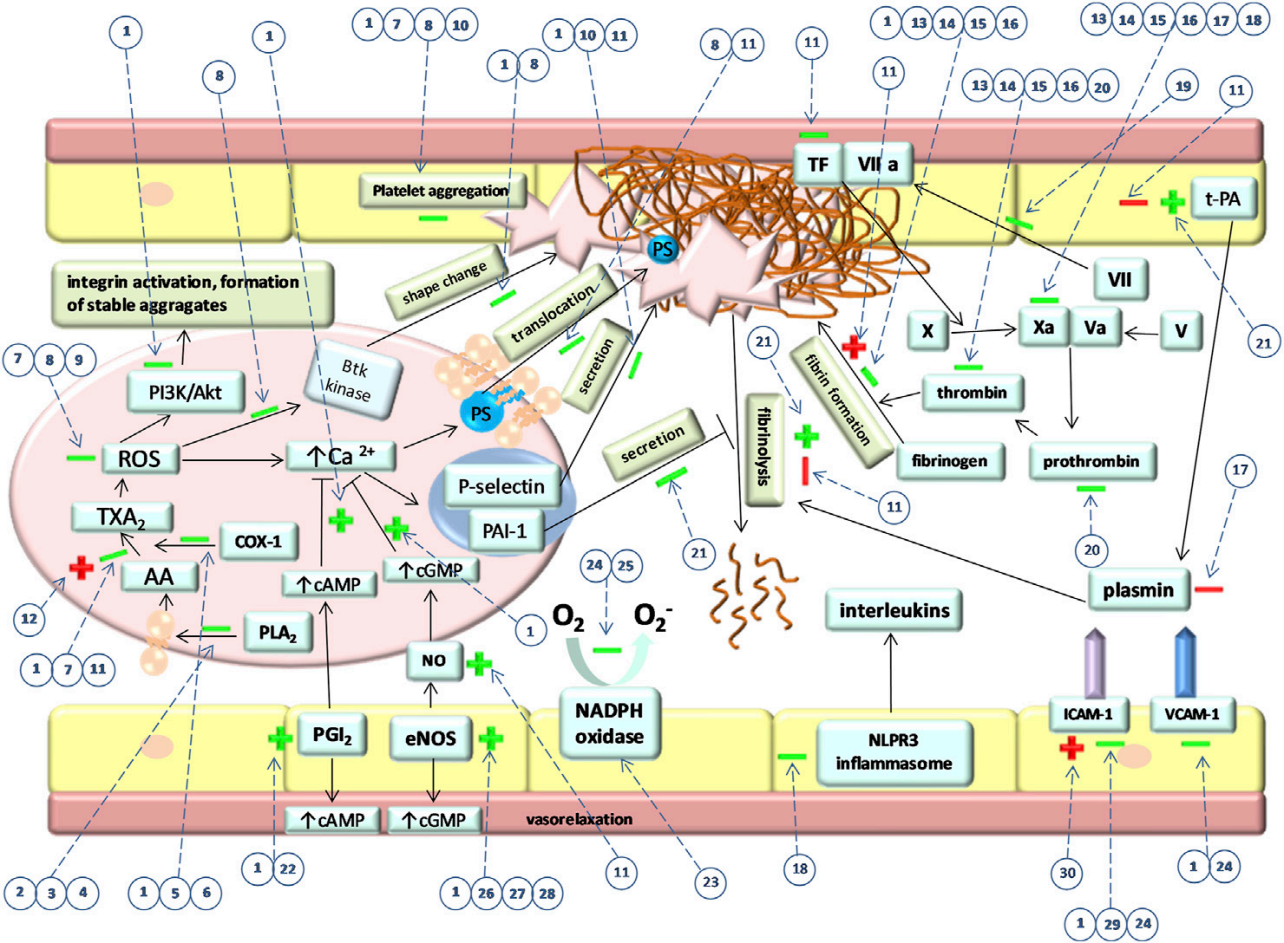
Main effects of tannins on hemostasis. “→” indicates activatory pathways and “˧” indicates inhibitory pathways that occur during hemostasis regulation. “+” and “–” indicate activatory and inhibitory effect of tannins on these pathways, respectively. Green color indicates favorable and red color indicates the unfavorable effect of tannins. Firstly, the picture shows the effect of tannins on particular signaling pathways in platelet which eventually also affect their shape change, PS translocation, secretion, and aggregation. Furthermore, the effect of tannins on coagulation factors and fibrin formation is shown. The influence of tannins on fibrinolysis and components that regulate this process is presented. The picture shows also tannins effect on endothelial NO and PGI_2_ synthesis as well as ICAM-1 and VCAM-1 expression. Numbers in circles represent specific compounds, fractions or extracts. Dotted lines indicate the processes affected by tannins. (1) PGG, (2) parameritannin A1, (3) aesculitannin B1, (4) oligomeric B-type procyanidins, (5) O-trigalloyl-beta-D-glucose, (6) isocorilagin, (7) pomegranate extract, (8) cinnamtannin 1B, (9) water–methanolic procyanidin fraction of the water–methanolic extract obtained from *Medemia argun* nuts, (10) cocoa procyanidins, (11) water-methanolic *Potentilla erecta* rhizome extract, (12) rugosin E, (13) pedunculagin, (14) tellimagrandin II, (15) casuariin, (16) 5-desgalloylstachyurin, (17) procyanidin fraction of an aqueous extract of *Brownea grandiceps* Jacq., (18) procyanidin B2, (19) hamamelitannin, (20) walnuts, (21) corilagin, (22) procyanidin-rich chocolate, (23) procyanidin fractions from grape pomace, (24) grape seeds procyanidin, (25) procyanidin fraction of a water–ethanolic extract from *Croton celtidifoliu*s, (26) 2,3-cis procyanidins (isolated from the ethyl acetate fraction of a methanolic extract from *Nelia meyeri* leaves (27) 2,3-trans procyanidins (isolated from the ethyl acetate fraction of a methanolic extract from *Salix* spp. Bark, (28) procyanidin C1 (29) procyanidin fraction of an acetone–water extract obtained from *Ribes nigrum*, (30) polyphenol fractions of acetone–water extracts obtained from *Rubus caesius* and *Rubus idaeus*.

Plant extracts, fractions, or compounds exhibit multidirectional effects in the hemostasis system. Among the compounds of plant origin, tannins can modify the activity of platelets, coagulation, the fibrinolysis system, and endothelium. Furthermore, they display thrombosis-inhibitory effect. Although most of the studies presented in this review have demonstrated the favorable effects of tannins on hemostasis, some undesirable effects were also reported. Due to of the low bioavailability of tannins and the potent activity of their metabolites, *in vitro* research works, which constitute the majority of studies cited in this review, do not provide reliable information on the activity of tannins *in vivo*. This paper highlights the fact that the activity of tannins could vary depending on the pathological state. Considering the key role of the gut microbiota in the formation of biologically active metabolites of tannins, it can be assumed that changes in the gut microflora during pathological conditions e.g., diabetes (Ma et al., 2020) could lead to the formation of different metabolites characterized by different biological activity. Another important issue is the changed pharmacokinetics of tannins under pathological state. After absorption to the blood, urolithins undergo glucuronidation and become inactive but during inflammation they are deconjugated and become free urolithins (Piwowarski et al., 2017). Therefore, future studies should aim at comparing the bioavailability and activity of tannins under normal and pathological conditions *in vivo.* Figure 2 illustrates the main effects of tannins on hemostasis.

## Glossary

6-keto-PGF1α: 6-keto prostaglandin F1α
AA: arachidonic acid
ADP: adenosine diphosphate
APTT: activated partial thromboplastin time
ASA: acetylsalicylic acid
CD39: ectonucleoside triphosphate diphosphohydrolase-1
COX: cyclooxygenase
eNOS: endothelial nitric oxide synthase
GP VI: glycoprotein VI
ICAM-1: intercellular adhesion molecule 1
IL: interleukin
LPS: liposaccharide
NADPH: nicotinamide adenine dinucleotide phosphate
NO: nitric oxide
**O**_**2**_^**−**^: superoxide anion
**ONOO** ^**−**^: peroxynitrate
PAI-1: plasminogen activator inhibitor-1
**PLA**_**2**_: phopsholipase A_2_
PGG: 1,2,3,4,6-pentagalloylglucose
**PGI**_**2**_: prostacyclin
PI3k: phosphatidylinositol 3-kinase
PLCβ: phospholipase β
PS: phosphatydilserine
PGs: prostanglandins
PT: partial thromboplastin time
ROS: reactive oxygen species
SIN-1: 3-morpholinosydnonimine
STZ: streptozotocin
TF: tissue factor
TNFα: tumor necrosis factor alpha
t-PA: tissue plasminogen activator
TT: thrombin time
**TXA**_**2**_: thromboxane A_2_
**TXB**_**2**_: thromboxane B_2_
u-PA: urokinse type plasminogen activator
VCAM-1: vascular cell adhesion molecule 1
vWF: von Willebrand factor

## References

[B1] Adamczyk B. Simon J. Kitunen V. Adamczyk S. Smolander A. (2017). Tannins and Their Complex Interaction with Different Organic Nitrogen Compounds and Enzymes: Old Paradigms versus Recent Advances. ChemistryOpen 6, 610–614. 10.1002/open.201700113 29046854 PMC5641916

[B2] Aldini G. Carini M. Piccoli A. Rossoni G. Facino R. M. (2003). Procyanidins from Grape Seeds Protect Endothelial Cells from Peroxynitrite Damage and Enhance Endothelium-dependent Relaxation in Human Artery: New Evidences for Cardio-protection. Life Sci. 73, 2883–2898. 10.1016/S0024-3205(03)00697-0 14511773

[B3] Álvarez E. Rodiño-Janeiro B. K. Jerez M. Ucieda-Somoza R. Núñez M. J. González-Juanatey J. R. (2012). Procyanidins from Grape Pomace Are Suitable Inhibitors of Human Endothelial NADPH Oxidase. J. Cel. Biochem. 113, 1386–1396. 10.1002/jcb.24011 22134950

[B4] Antonioli L. Pacher P. Vizi E. S. Haskó G. (2013). CD39 and CD73 in Immunity and Inflammation. Trends Mol. Med. 19, 355–367. 10.1016/j.molmed.2013.03.005 23601906 PMC3674206

[B5] Aron P. M. Kennedy J. A. (2008). Flavan-3-ols: Nature, Occurrence and Biological Activity. Mol. Nutr. Food Res. 52, 79–104. 10.1002/mnfr.200700137 18081206

[B6] Aviram M. Volkova N. Coleman R. Dreher M. Reddy M. K. Ferreira D. (2008). Pomegranate Phenolics from the Peels, Arils, and Flowers Are Antiatherogenic: Studies *In Vivo* in Atherosclerotic Apolipoprotein E-Deficient (E 0) Mice and *In Vitro* in Cultured Macrophages and Lipoproteins. J. Agric. Food Chem. 56, 1148–1157. 10.1021/jf071811q 18173244

[B7] Ben Amor N. Bouaziz A. Romera-Castillo C. Salido S. Linares-Palomino P. J. Bartegi A. (2007). Characterization of the Intracellular Mechanisms Involved in the Antiaggregant Properties of Cinnamtannin B-1 from bay wood in Human Platelets. J. Med. Chem. 50, 3937–3944. 10.1021/jm070508d 17602466

[B8] Ben Amor N. Pariente J. A. Salido G. M. Bartegi A. Rosado J. A. (2006). Caspases 3 and 9 Are Translocated to the Cytoskeleton and Activated by Thrombin in Human Platelets. Evidence for the Involvement of PKC and the Actin Filament Polymerization. Cell. Signal. 18, 1252–1261. 10.1016/j.cellsig.2005.10.002 16300929

[B9] Beretta G. Rossoni G. Santagati N. A. Facino R. M. (2009). Anti-ischemic Activity and Endothelium-dependent Vasorelaxant Effect of Hydrolysable Tannins from the Leaves of *Rhus Coriaria* (Sumac) in Isolated Rabbit Heart and Thoracic Aorta. Planta Med. 75, 1482–1488. 10.1055/s-0029-1185797 19548191

[B10] Bijak M. Ponczek M. B. Nowak P. (2014). Polyphenol Compounds Belonging to Flavonoids Inhibit Activity of Coagulation Factor X. Int. J. Biol. Macromol. 65, 129–135. 10.1016/j.ijbiomac.2014.01.023 24444877

[B11] Blache D. Durand P. Prost M. Loreau N. (2002). (+)-Catechin Inhibits Platelet Hyperactivity Induced by an Acute Iron Load *In Vivo* . Free Radic. Biol. Med. 33, 1670–1680. 10.1016/S0891-5849(02)01139-5 12488135

[B12] Bock P. E. Srinivasan K. R. Shore J. D. (1981). Activation of Intrinsic Blood Coagulation by Ellagic Acid: Insoluble Ellagic Acid-Metal Ion Complexes Are the Activating Species. Biochemistry 20, 7258–7266. 10.1021/bi00528a032 6797471

[B13] Borisova M. P. Kataev A. A. Sivozhelezov V. S. (2019). Action of Tannin on Cellular Membranes: Novel Insights from Concerted Studies on Lipid Bilayers and Native Cells. Biochim. Biophys. Acta Biomembr 1861, 1103–1111. 10.1016/j.bbamem.2019.03.017 30926363

[B14] Bouaziz A. Romera-Castillo C. Salido S. Linares-Palomino P. J. Altarejos J. Bartegi A. (2007a). Cinnamtannin B-1 from bay wood Exhibits Antiapoptotic Effects in Human Platelets. Apoptosis 12, 489–498. 10.1007/s10495-006-0014-z 17195094

[B15] Bouaziz A. Salido S. Linares-Palomino P. J. Sanchez A. Altarejos J. Bartegi A. (2007b). Cinnamtannin B-1 from bay wood Reduces Abnormal Intracellular Ca2+ Homeostasis and Platelet Hyperaggregability in Type 2 Diabetes Mellitus Patients. Arch. Biochem. Biophys. 457, 235–242. 10.1016/j.abb.2006.10.020 17118329

[B16] Byun E. B. Sung N. Y. Yang M. S. Song D. S. Byun E. H. Kim J. K. (2014). Procyanidin C1 Causes Vasorelaxation through Activation of the Endothelial NO/cGMP Pathway in Thoracic Aortic Rings. J. Med. Food 17, 742–748. 10.1089/jmf.2013.2978 24971771 PMC4098072

[B17] Byun M. W. (2012). Effect of Procyanidin C1 on Nitric Oxide Production and Hyperpolarization through Ca(2+)-dependent Pathway in Endothelial Cells. J. Med. Food 15, 1032–1037. 10.1089/jmf.2012.2297 23057780

[B18] Campana P. R. Braga F. C. Cortes S. F. (2009). Endothelium-dependent Vasorelaxation in Rat Thoracic Aorta by *Mansoa Hirsuta* D.C. Phytomedicine 16, 456–461. 10.1016/j.phymed.2008.09.007 19019646

[B19] Cao Y. Himmeldirk K. B. Qian Y. Ren Y. Malki A. Chen X. (2014). Biological and Biomedical Functions of Penta-O-Galloyl-D-Glucose and its Derivatives. J. Nat. Med. 68, 465–472. 10.1007/s11418-014-0823-2 24532420

[B20] Carnevale R. Loffredo L. Nocella C. Bartimoccia S. Bucci T. De Falco E. (2014). Epicatechin and Catechin Modulate Endothelial Activation Induced by Platelets of Patients with Peripheral Artery Disease. Oxid. Med. Cel. Longev. 2014, 691015. 10.1155/2014/691015 PMC4142301 25180068

[B21] Chang S.-S. Lee V. S. Y. Tseng Y.-L. Chang K.-C. Chen K.-B. Chen Y.-L. (2012). Gallic Acid Attenuates Platelet Activation and Platelet-Leukocyte Aggregation: Involving Pathways of Akt and GSK3β. Evidence-Based Complement. Altern. Med. 2012, 1–8. 10.1155/2012/683872 PMC3395410 22811749

[B22] Chen J. J. Lee H. H. Shih C. D. Liao C. H. Chen I. S. Chou T. H. (2007). New Dihydrochalcones and Anti-platelet Aggregation Constituents from the Leaves of *Muntingia calabura* . Planta Med. 73, 572–577. 10.1055/s-2007-967196 17516329

[B23] Cheng X. Zhang J. Jing H. Qi Y. Yan T. Wu B. (2020). Pharmacokinetic Differences of Grape Seed Procyanidins According to the Gavage Administration between Normal Rats and Alzheimer's Disease Rats. Cpa 17, 119–128. 10.2174/1573412915666190916161225

[B24] Ciciliano J. C. Sakurai Y. Myers D. R. Fay M. E. Hechler B. Meeks S. (2015). Resolving the Multifaceted Mechanisms of the Ferric Chloride Thrombosis Model Using an Interdisciplinary Microfluidic Approach. Blood 126, 817–824. 10.1182/blood-2015-02-628594 25931587 PMC4528067

[B25] Côrtes S. F. Valadares Y. M. de Oliveira A. B. Lemos V. S. Barbosa M. P. Braga F. C. (2002). Mechanism of Endothelium-dependent Vasodilation Induced by a Proanthocyanidin-Rich Fraction from *Ouratea Semiserrata* . Planta Med. 68, 412–415. 10.1055/s-2002-32079 12058316

[B26] DalBó S. Goulart S. Horst H. Pizzolatti M. G. Ribeiro-do-Valle R. M. (2008). Activation of Endothelial Nitric Oxide Synthase by Proanthocyanidin-Rich Fraction from *Croton Celtidifolius* (Euphorbiaceae): Involvement of Extracellular Calcium Influx in Rat Thoracic Aorta. J. Pharmacol. Sci. 107, 181–189. 10.1254/jphs.FP0072385 18566521

[B27] De Curtis A. Amore C. Donati M. B. De Gaetano G. Iacoviello L. (2003). A Procyanidin Extract Prolongs Bleeding Time but Does Not Prevent Thrombosis in Rats. J. Thromb. Haemost. 1, 199–200. 10.1046/j.1538-7836.2003.00018.x 12871565

[B28] Dong H. Chen S. X. Kini R. M. Xu H. X. (1998). Effects of Tannins from *Geum Japonicum* on the Catalytic Activity of Thrombin and Factor Xa of Blood Coagulation cascade. J. Nat. Prod. 61, 1356–1360. 10.1021/np9801458 9834152

[B29] Dudzinska D. Luzak B. Boncler M. Rywaniak J. Sosnowska D. Podsedek A. (2014). CD39/NTPDase-1 Expression and Activity in Human Umbilical Vein Endothelial Cells Are Differentially Regulated by Leaf Extracts from *Rubus Caesius* and *Rubus Idaeus* . Cell. Mol. Biol. Lett. 19, 361–380. 10.2478/s11658-014-0202-8 25034034 PMC6275654

[B30] Espín J. C. Larrosa M. García-Conesa M. T. Tomás-Barberán F. (2013). Biological Significance of Urolithins, the Gut Microbial Ellagic Acid-Derived Metabolites: the Evidence So Far. Evidence-Based Complement. Altern. Med. 2013, 1–15. 10.1155/2013/270418 PMC3679724 23781257

[B31] Fine A. M. (2000). Oligomeric Proanthocyanidin Complexes: History, Structure, and Phytopharmaceutical Applications. Altern. Med. Rev. 5, 144–151. Available at: http://www.ncbi.nlm.nih.gov/pubmed/10767669 . 10767669

[B32] Flaumenhaft R. (2017). Stressed Platelets ASK1 for a MAPK. Blood 129, 1066–1068. 10.1182/blood-2017-01-760546 28254824 PMC5374735

[B33] Fraga-Corral M. Otero P. Cassani L. Echave J. Garcia-Oliveira P. Carpena M. (2021). Traditional Applications of Tannin Rich Extracts Supported by Scientific Data: Chemical Composition, Bioavailability and Bioaccessibility. Foods 10, 251. 10.3390/foods10020251 33530516 PMC7912241

[B34] Furie B. Furie B. C. (2007). *In Vivo* thrombus Formation. J. Thromb. Haemost. 5, 12–17. 10.1111/j.1538-7836.2007.02482.x 17635703

[B35] Furuuchi R. Sakai H. Hirokawa N. Watanabe Y. Yokoyama T. Hirayama M. (2012). Antihypertensive Effect of Boysenberry Seed Polyphenols on Spontaneously Hypertensive Rats and Identification of Orally Absorbable Proanthocyanidins with Vasorelaxant Activity. Biosci. Biotechnol. Biochem. 76, 1694–1701. 10.1271/bbb.120302 22972352

[B36] Gale A. J. (2011). Continuing Education Course #2: Current Understanding of Hemostasis. Toxicol. Pathol. 39, 273–280. 10.1177/0192623310389474 21119054 PMC3126677

[B37] Garbacki N. Kinet M. Nusgens B. Desmecht D. Damas J. (2005). Proanthocyanidins, from *Ribes Nigrum* Leaves, Reduce Endothelial Adhesion Molecules ICAM-1 and VCAM-1. J. Inflamm. (Lond) 2, 9. 10.1186/1476-9255-2-9 16091140 PMC1198248

[B38] González-Sarrías A. García-Villalba R. Núñez-Sánchez M. Á. Tomé-Carneiro J. Zafrilla P. Mulero J. (2015). Identifying the Limits for Ellagic Acid Bioavailability: A Crossover Pharmacokinetic Study in Healthy Volunteers after Consumption of Pomegranate Extracts. J. Funct. Foods 19, 225–235. 10.1016/j.jff.2015.09.019

[B39] Goto H. Shimada Y. Akechi Y. Kohta K. Hattori M. Terasawa K. (1996). Endothelium-dependent Vasodilator Effect of Extract Prepared from the Roots of *Paeonia Lactiflora* on Isolated Rat Aorta. Planta Med. 62, 436–439. 10.1055/s-2006-957934 8923810

[B40] Guidetti G. F. Canobbio I. Torti M. (2015). PI3K/Akt in Platelet Integrin Signaling and Implications in Thrombosis. Adv. Biol. Regul. 59, 36–52. 10.1016/j.jbior.2015.06.001 26159296

[B41] Hagerman A. E. (1989). Chemistry of Tannin-Protein Complexation. Chemistry And Significance Of Condensed Tannins. Boston, MA: Springer US, 323–333. 10.1007/978-1-4684-7511-1_20

[B42] Harlan J. M. Harker L. A. (1981). Hemostasis, Thrombosis, and Thromboembolic Disorders. The Role of Arachidonic Acid Metabolites in Platelet-Vessel wall Interactions. Med. Clin. North. Am. 65, 855–880. 10.1016/S0025-7125(16)31502-4 6792433

[B43] Harper M. T. Poole A. W. (2013). Chloride Channels Are Necessary for Full Platelet Phosphatidylserine Exposure and Procoagulant Activity. Cell Death Dis 4, e969. 10.1038/cddis.2013.495 24357800 PMC3877565

[B44] Hayashi T. Mogami H. Murakami Y. Nakamura T. Kanayama N. Konno H. (2008). Real-time Analysis of Platelet Aggregation and Procoagulant Activity during Thrombus Formation *In Vivo* . Pflugers Arch. 456, 1239–1251. 10.1007/s00424-008-0466-9 18553102

[B45] Hoffman M. (2001). Remodeling the Blood Coagulation cascade. J. Thromb. Thrombolysis 16, 17–20. 10.1023/B:THRO.0000014588.95061.28 14760207

[B46] Hort M. A. Straliotto M. R. Duz M. S. Netto P. M. Souza C. B. Schulz T. (2012). Cardioprotective Effects of a Proanthocyanidin-Rich Fraction from *Croton Celtidifolius* Baill: Focus on Atherosclerosis. Food Chem. Toxicol. 50, 3769–3775. 10.1016/j.fct.2012.07.050 22884763

[B47] Jeon W. K. Lee J. H. Kim H. K. Lee A. Y. Lee S. O. Kim Y. S. (2006). Anti-platelet Effects of Bioactive Compounds Isolated from the Bark of *Rhus Verniciflua* Stokes. J. Ethnopharmacol. 106, 62–69. 10.1016/j.jep.2005.12.015 16431050

[B48] Ji M. S. Piao X. L. Jin Y. L. Park R. D. (2005). Anticoagulant 1,2,3,4,6-Pentagalloyl-Beta-D-Glucopyranose Isolated from geranium (Pelargonium Inquinans Ait). Arch. Pharm. Res. 28, 1037–1041. 10.1007/BF02977398 16212234

[B49] Jiamboonsri P. Pithayanukul P. Bavovada R. Leanpolchareanchai J. Yin T. Gao S. (2015). Factors Influencing Oral Bioavailability of Thai Mango Seed Kernel Extract and its Key Phenolic Principles. Molecules 20, 21254–21273. 10.3390/molecules201219759 26633325 PMC6332320

[B50] Jiang Y. Li Y. Ding Y. Dai X. Ma X. Bao L. (2015). Grape Seed Proanthocyanidin Extracts Prevent High Glucose-Induced Endothelia Dysfunction via PKC and NF-Κb Inhibition. Biosci. Biotechnol. Biochem. 79, 1493–1503. 10.1080/09168451.2014.991679 26072928

[B51] Kang D. G. Moon M. K. Choi D. H. Lee J. K. Kwon T. O. Lee H. S. (2005). Vasodilatory and Anti-inflammatory Effects of the 1,2,3,4,6-Penta-O-Galloyl-Beta-D-Glucose (PGG) via a Nitric Oxide-cGMP Pathway. Eur. J. Pharmacol. 524, 111–119. 10.1016/j.ejphar.2005.08.061 16253226

[B52] Kaufeld A. M. Pertz H. H. Kolodziej H. (2013). 2,3-cis-procyanidins Elicit Endothelium-dependent Relaxation in Porcine Coronary Arteries via Activation of the PI3/Akt Kinase Signaling Pathway. J. Agric. Food Chem. 61, 9609–9616. 10.1021/jf402460m 24032351

[B53] Kaufeld A. M. Pertz H. H. Kolodziej H. (2014). A Chemically Defined 2,3-trans Procyanidin Fraction from Willow Bark Causes Redox-Sensitive Endothelium-dependent Relaxation in Porcine Coronary Arteries. J. Nat. Prod. 77, 1607–1614. 10.1021/np500177u 24957134

[B54] Khanbabaee K. van Ree T. (2001). Tannins: Classification and Definition. Nat. Prod. Rep. 18, 641–649. 10.1039/B101061L 11820762

[B55] Kim S. H. Kang K. W. Kim K. W. Kim N. D. (2000). Procyanidins in Crataegus Extract Evoke Endothelium-dependent Vasorelaxation in Rat Aorta. Life Sci. 67, 121–131. 10.1016/S0024-3205(00)00608-1 10901280

[B56] Kiss A. K. Piwowarski J. P. (2019). Ellagitannins, Gallotannins and Their Metabolites- the Contribution to the Anti-inflammatory Effect of Food Products and Medicinal Plants. Curr. Med. Chem. 25, 4946–4967. 10.2174/0929867323666160919111559 27655073

[B57] Lagoutte-Renosi J. Allemand F. Ramseyer C. Rabani V. Davani S. (2021). Influence of Antiplatelet Agents on the Lipid Composition of Platelet Plasma Membrane: A Lipidomics Approach with Ticagrelor and its Active Metabolite. Int. J. Mol. Sci. 22, 1432. 10.3390/ijms22031432 33572690 PMC7866994

[B58] Lambert J. D. Yennawar N. Gu Y. Elias R. J. (2012). Inhibition of Secreted Phospholipase A2 by Proanthocyanidins: a Comparative Enzymological and *In Silico* Modeling Study. J. Agric. Food Chem. 60, 7417–7420. 10.1021/jf302308k 22779786 PMC3740037

[B59] Le Bourvellec C. Renard C. M. (2012). Interactions between Polyphenols and Macromolecules: Quantification Methods and Mechanisms. Crit. Rev. Food Sci. Nutr. 52, 213–248. 10.1080/10408398.2010.499808 22214442

[B60] Lee J. J. Cho W. K. Kwon H. Gu M. Ma J. Y. (2014). Galla Rhois Exerts its Antiplatelet Effect by Suppressing ERK1/2 and PLCβ Phosphorylation. Food Chem. Toxicol. 69, 94–101. 10.1016/j.fct.2014.03.032 24685825

[B61] Levy J. H. Szlam F. Wolberg A. S. Winkler A. (2014). Clinical Use of the Activated Partial Thromboplastin Time and Prothrombin Time for Screening: a Review of the Literature and Current Guidelines for Testing. Clin. Lab. Med. 34, 453–477. 10.1016/j.cll.2014.06.005 25168937

[B62] Liang Y. Li J. Lin Q. Huang P. Zhang L. Wu W. (2017). Research Progress on Signaling Pathway-Associated Oxidative Stress in Endothelial Cells. Oxid. Med. Cel. Longev. 2017, 7156941–7156948. 10.1155/2017/7156941 PMC5414589 28503253

[B63] Litvinov R. I. Weisel J. W. (2017). Role of Red Blood Cells in Haemostasis and Thrombosis. ISBT Sci. Ser. 12, 176–183. 10.1111/voxs.12331 28458720 PMC5404239

[B64] López de Felipe F. de las Rivas B. MuÃ±oz R. (2014). Bioactive Compounds Produced by Gut Microbial Tannase: Implications for Colorectal Cancer Development. Front. Microbiol. 5. 10.3389/fmicb.2014.00684 PMC4257100 25538697

[B65] Lu Y. Bennick A. (1998). Interaction of Tannin with Human Salivary Proline-Rich Proteins. Arch. Oral Biol. 43, 717–728. 10.1016/S0003-9969(98)00040-5 9783826

[B66] Luzak B. Boncler M. Rywaniak J. Dudzinska D. Rozalski M. Krajewska U. (2014). Extract from *Ribes Nigrum* Leaves *In Vitro* Activates Nitric Oxide Synthase (eNOS) and Increases CD39 Expression in Human Endothelial Cells. J. Physiol. Biochem. 70, 1007–1019. 10.1007/s13105-014-0370-z 25407137 PMC4254183

[B67] Ma C. Yao Y. Yue Q. X. Zhou X. W. Yang P. Y. Wu W. Y. (2011). Differential Proteomic Analysis of Platelets Suggested Possible Signal Cascades Network in Platelets Treated with Salvianolic Acid B. PLoS One 6, e14692. 10.1371/journal.pone.0014692 21379382 PMC3040754

[B68] Ma Q. Li Y. Wang J. Li P. Duan Y. Dai H. (2020). Investigation of Gut Microbiome Changes in Type 1 Diabetic Mellitus Rats Based on High-Throughput Sequencing. Biomed. Pharmacother. 124, 109873. 10.1016/j.biopha.2020.109873 31986412

[B69] Magos G. A. Mateos J. C. Páez E. Fernández G. Lobato C. Márquez C. (2008). Hypotensive and Vasorelaxant Effects of the Procyanidin Fraction from *Guazuma ulmifolia* Bark in Normotensive and Hypertensive Rats. J. Ethnopharmacol. 117, 58–68. 10.1016/j.jep.2008.01.015 18314282

[B70] Magwenzi S. Woodward C. Wraith K. S. Aburima A. Raslan Z. Jones H. (2015). Oxidized LDL Activates Blood Platelets through CD36/NOX2-Mediated Inhibition of the cGMP/protein Kinase G Signaling cascade. Blood 125, 2693–2703. 10.1182/blood-2014-05-574491 25710879 PMC4408294

[B71] Marcinczyk N. Gołaszewska A. Gromotowicz-Poplawska A. Misztal T. Strawa J. Tomczyk M. (2021a). Multidirectional Effects of Tormentil Extract on Hemostasis in Experimental Diabetes. Front. Pharmacol. 12, 682987. 10.3389/fphar.2021.682987 34025439 PMC8131833

[B72] Marcinczyk N. Jarmoc D. Leszczynska A. Zakrzeska A. Kramkowski K. Strawa J. (2017). Antithrombotic Potential of Tormentil Extract in Animal Models. Front. Pharmacol. 8, 534. 10.3389/fphar.2017.00534 28860991 PMC5559472

[B73] Marcinczyk N. Misztal T. Gromotowicz-Poplawska A. Zebrowska A. Rusak T. Radziwon P. (2021b). Utility of Platelet Endothelial Cell Adhesion Molecule 1 in the Platelet Activity Assessment in Mouse and Human Blood. Int. J. Mol. Sci. 22, 9611. 10.3390/ijms22179611 34502520 PMC8431756

[B74] Matsui T. Korematsu S. Byun E. B. Nishizuka T. Ohshima S. Kanda T. (2009). Apple Procyanidins Induced Vascular Relaxation in Isolated Rat Aorta through NO/cGMP Pathway in Combination with Hyperpolarization by Multiple K+ Channel Activations. Biosci. Biotechnol. Biochem. 73, 2246–2251. 10.1271/bbb.90334 19809179

[B75] Mattiello T. Trifirò E. Jotti G. S. Pulcinelli F. M. (2009). Effects of Pomegranate Juice and Extract Polyphenols on Platelet Function. J. Med. Food 12, 334–339. 10.1089/jmf.2007.0640 19459734

[B76] Mazzanti L. Rabini R. A. Fumelli P. Martarelli D. Staffolani R. Salvolini E. (1997). Altered Platelet Membrane Dynamic Properties in Type 1 Diabetes. Diabetes 46, 2069–2074. 10.2337/diab.46.12.2069 9392498

[B77] Mega J. L. Simon T. (2015). Pharmacology of Antithrombotic Drugs: An Assessment of Oral Antiplatelet and Anticoagulant Treatments. Lancet 386, 281–291. 10.1016/S0140-6736(15)60243-4 25777662

[B78] Mekhfi H. ElHaouari M. Bnouham M. Aziz M. Ziyyat A. Legssyer A. (2006). Effects of Extracts and Tannins from *Arbutus unedo* Leaves on Rat Platelet Aggregation. Phytother Res. 20, 135–139. 10.1002/ptr.1822 16444667

[B79] Mertens-Talcott S. U. Jilma-Stohlawetz P. Rios J. Hingorani L. Derendorf H. (2006). Absorption, Metabolism, and Antioxidant Effects of Pomegranate (*Punica Granatum* L.) Polyphenols after Ingestion of a Standardized Extract in Healthy Human Volunteers. J. Agric. Food Chem. 54, 8956–8961. 10.1021/jf061674h 17090147

[B80] Mitchell J. A. Ali F. Bailey L. Moreno L. Harrington L. S. (2008). Role of Nitric Oxide and Prostacyclin as Vasoactive Hormones Released by the Endothelium. Exp. Physiol. 93, 141–147. 10.1113/expphysiol.2007.038588 17965142

[B81] Morel A. Hamed A. I. Oleszek W. Stochmal A. Głowacki R. Olas B. (2014). Protective Action of Proanthocyanidin Fraction from *Medemia argun* Nuts against Oxidative/nitrative Damages of Blood Platelet and Plasma Components. Platelets 25, 75–80. 10.3109/09537104.2013.769511 23560434

[B82] Murphy K. J. Chronopoulos A. K. Singh I. Francis M. A. Moriarty H. Pike M. J. (2003). Dietary Flavanols and Procyanidin Oligomers from cocoa (*Theobroma Cacao*) Inhibit Platelet Function. Am. J. Clin. Nutr. 77, 1466–1473. 10.1093/ajcn/77.6.1466 12791625

[B83] Nanetti L. Vignini A. Raffaelli F. Moroni C. Silvestrini M. Provinciali L. (2008). Platelet Membrane Fluidity and Na+/K+ ATPase Activity in Acute Stroke. Brain Res. 1205, 21–26. 10.1016/j.brainres.2008.02.005 18343354

[B84] Nergiz-Ünal R. Kuijpers M. J. de Witt S. M. Heeneman S. Feijge M. A. Garcia Caraballo S. C. (2013). Atheroprotective Effect of Dietary walnut Intake in ApoE-Deficient Mice: Involvement of Lipids and Coagulation Factors. Thromb. Res. 131, 411–417. 10.1016/j.thromres.2013.01.003 23347681

[B85] Novakovic A. Marinko M. Jankovic G. Stojanovic I. Milojevic P. Nenezic D. (2017). Endothelium-dependent Vasorelaxant Effect of Procyanidin B2 on Human Internal Mammary Artery. Eur. J. Pharmacol. 807, 75–81. 10.1016/j.ejphar.2017.04.015 28414054

[B86] Ohmori T. Yatomi Y. Asazuma N. Satoh K. Ozaki Y. (2000). Involvement of Proline-Rich Tyrosine Kinase 2 in Platelet Activation: Tyrosine Phosphorylation Mostly Dependent on alphaIIbbeta3 Integrin and Protein Kinase C, Translocation to the Cytoskeleton and Association with Shc through Grb2. Biochem. J. 347, 561–569. 10.1042/0264-6021:3470561 10749687 PMC1220990

[B87] Okudan N. Barışkaner H. Gökbel H. Sahin A. S. Belviranlı M. Baysal H. (2011). The Effect of Supplementation of Grape Seed Proanthocyanidin Extract on Vascular Dysfunction in Experimental Diabetes. J. Med. Food 14, 1298–1302. 10.1089/jmf.2010.0030 21663473

[B88] Ou K. Gu L. (2014). Absorption and Metabolism of Proanthocyanidins. J. Funct. Foods 7, 43–53. 10.1016/j.jff.2013.08.004

[B89] Pereira B. Brazón J. Rincón M. Vonasek E. (2017). Browplasminin, a Condensed Tannin with Anti-plasmin Activity Isolated from an Aqueous Extract of Brownea Grandiceps Jacq. Flowers. J. Ethnopharmacol 198, 282–290. 10.1016/j.jep.2017.01.012 28089737

[B90] Perveen R. Funk K. Thuma J. Wulf Ridge S. Cao Y. Akkerman J. W. (2011). A Novel Small Molecule 1,2,3,4,6-Penta-O-Galloyl-α-D-Glucopyranose Mimics the Antiplatelet Actions of Insulin. PLoS One 6, e26238. 10.1371/journal.pone.0026238 22073153 PMC3206812

[B91] Pfundstein B. Haubner R. Würtele G. Gehres N. Ulrich C. M. Owen R. W. (2014). Pilot walnut Intervention Study of Urolithin Bioavailability in Human Volunteers. J. Agric. Food Chem. 62, 10264–10273. 10.1021/jf5040652 25275327

[B92] Pietraforte D. Vona R. Marchesi A. de Jacobis I. T. Villani A. Del Principe D. (2014). Redox Control of Platelet Functions in Physiology and Pathophysiology. Antioxid. Redox Signal. 21, 177–193. 10.1089/ars.2013.5532 24597688

[B93] Pinna C. Morazzoni P. Sala A. (2017). Proanthocyanidins from *Vitis vinifera* Inhibit Oxidative Stress-Induced Vascular Impairment in Pulmonary Arteries from Diabetic Rats. Phytomedicine 25, 39–44. 10.1016/j.phymed.2016.12.015 28190469

[B94] Piwowarski J. P. Granica S. Stefańska J. Kiss A. K. (2016). Differences in Metabolism of Ellagitannins by Human Gut Microbiota *Ex Vivo* Cultures. J. Nat. Prod. 79, 3022–3030. 10.1021/acs.jnatprod.6b00602 28006907

[B95] Piwowarski J. P. Stanisławska I. Granica S. Stefańska J. Kiss A. K. (2017). Phase II Conjugates of Urolithins Isolated from Human Urine and Potential Role of β-Glucuronidases in Their Disposition. Drug Metab. Dispos. 45, 657–665. 10.1124/dmd.117.075200 28283501

[B96] Poznyak A. V. Nikiforov N. G. Markin A. M. Kashirskikh D. A. Myasoedova V. A. Gerasimova E. V. (2020). Overview of OxLDL and its Impact on Cardiovascular Health: Focus on Atherosclerosis. Front. Pharmacol. 11, 613780. 10.3389/fphar.2020.613780 33510639 PMC7836017

[B97] Qiu H. Zhang L. Zhu M. Zhang M. Chen J. Feng L. (2017). Capture of Anti-coagulant Active Ingredients from Moutan Cortex by Platelet Immobilized Chromatography and Evaluation of Anticoagulant Activity in Rats. Biomed. Pharmacother. 95, 235–244. 10.1016/j.biopha.2017.08.051 28843912

[B98] Sakariassen K. S. Orning L. Turitto V. T. (2015). The Impact of Blood Shear Rate on Arterial Thrombus Formation. Future Sci. OA 1, FSO30. 10.4155/fso.15.28 28031903 PMC5137878

[B99] Sano A. Yamakoshi J. Tokutake S. Tobe K. Kubota Y. Kikuchi M. (2003). Procyanidin B1 Is Detected in Human Serum after Intake of Proanthocyanidin-Rich Grape Seed Extract. Biosci. Biotechnol. Biochem. 67, 1140–1143. 10.1271/bbb.67.1140 12834296

[B100] Sano T. Oda E. Yamashita T. Naemura A. Ijiri Y. Yamakoshi J. (2005). Anti-thrombotic Effect of Proanthocyanidin, a Purified Ingredient of Grape Seed. Thromb. Res. 115, 115–121. 10.1016/j.thromres.2004.07.015 15567462

[B101] Sawa Y. Ueki T. Hata M. Iwasawa K. Tsuruga E. Kojima H. (2008). LPS-induced IL-6, IL-8, VCAM-1, and ICAM-1 Expression in Human Lymphatic Endothelium. J. Histochem. Cytochem. 56, 97–109. 10.1369/jhc.7A7299.2007 17938282 PMC2324174

[B102] Scalbert A. Morand C. Manach C. Rémésy C. (2002). Absorption and Metabolism of Polyphenols in the Gut and Impact on Health. Biomed. Pharmacother. 56, 276–282. 10.1016/S0753-3322(02)00205-6 12224598

[B103] Schramm D. D. Wang J. F. Holt R. R. Ensunsa J. L. Gonsalves J. L. Lazarus S. A. (2001). Chocolate Procyanidins Decrease the Leukotriene-Prostacyclin Ratio in Humans and Human Aortic Endothelial Cells. Am. J. Clin. Nutr. 73, 36–40. 10.1093/ajcn/73.1.36 11124747

[B104] Shen Z. Q. Dong Z. J. Peng H. Liu J. K. (2003). Modulation of PAI-1 and tPA Activity and Thrombolytic Effects of Corilagin. Planta Med. 69, 1109–1112. 10.1055/s-2003-45191 14750026

[B105] Sieniawska E. (2015). Activities of Tannins--From *In Vitro* Studies to Clinical Trials. Nat. Prod. Commun. 10, 1877–1884. 10.1177/1934578x1501001118 26749816

[B106] Sójka M. Janowski M. Grzelak-Błaszczyk K. (2019). Stability and Transformations of Raspberry (*Rubus Idaeus* L.) Ellagitannins in Aqueous Solutions. Eur. Food Res. Technol. 245, 1113–1122. 10.1007/s00217-018-3212-3

[B107] Spigoni V. Mena P. Cito M. Fantuzzi F. Bonadonna R. C. Brighenti F. (2016). Effects on Nitric Oxide Production of Urolithins, Gut-Derived Ellagitannin Metabolites, in Human Aortic Endothelial Cells. Molecules 21, 1009. 10.3390/molecules21081009 PMC6274502 27490528

[B108] Stone J. Hangge P. Albadawi H. Wallace A. Shamoun F. Knuttien M. G. (2017). Deep Vein Thrombosis: Pathogenesis, Diagnosis, and Medical Management. Cardiovasc. Diagn. Ther. 7, S276–S284. 10.21037/cdt.2017.09.01 29399531 PMC5778510

[B109] Stoupi S. Williamson G. Viton F. Barron D. King L. J. Brown J. E. (2010). *In Vivo* bioavailability, Absorption, Excretion, and Pharmacokinetics of [14C]procyanidin B2 in Male Rats. Drug Metab. Dispos. 38, 287–291. 10.1124/dmd.109.030304 19910517

[B110] Sun Q. A. Runge M. S. Madamanchi N. R. (2016). Oxidative Stress, NADPH Oxidases, and Arteries. Hamostaseologie 36, 77–88. 10.5482/HAMO-14-11-0076 25649240 PMC5625353

[B111] Teng C. M. Kang Y. F. Chang Y. L. Ko F. N. Yang S. C. Hsu F. L. (1997). ADP-mimicking Platelet Aggregation Caused by Rugosin E, an Ellagitannin Isolated from *Rosa Rugosa* Thunb. Thromb. Haemost. 77, 555–561. 10.1055/s-0038-1656005 9066010

[B112] Tomás-Barberán F. A. García-Villalba R. González-Sarrías A. Selma M. V. Espín J. C. (2014). Ellagic Acid Metabolism by Human Gut Microbiota: Consistent Observation of Three Urolithin Phenotypes in Intervention Trials, Independent of Food Source, Age, and Health Status. J. Agric. Food Chem. 62, 6535–6538. 10.1021/jf5024615 24976365

[B113] Tomás-Barberán F. A. González-Sarrías A. García-Villalba R. Núñez-Sánchez M. A. Selma M. V. García-Conesa M. T. (2017). Urolithins, the rescue of "old" Metabolites to Understand A "new" Concept: Metabotypes as a Nexus Among Phenolic Metabolism, Microbiota Dysbiosis, and Host Health Status. Mol. Nutr. Food Res. 61, 1500901. 10.1002/mnfr.201500901 27158799

[B114] Tousoulis D. Kampoli A. M. Tentolouris C. Papageorgiou N. Stefanadis C. (2012). The Role of Nitric Oxide on Endothelial Function. Curr. Vasc. Pharmacol. 10, 4–18. 10.2174/157016112798829760 22112350

[B115] Undas A. (2017). “Determination of Fibrinogen and Thrombin Time (TT),” in ”Hemostasis And Thrombosis: Methods and Protocols. Editors Favaloro E. J. Lippi G. (New York, NY: Springer New York), 105–110. 10.1007/978-1-4939-7196-1_8 28804822

[B116] van den Oever I. A. Raterman H. G. Nurmohamed M. T. Simsek S. (2010). Endothelial Dysfunction, Inflammation, and Apoptosis in Diabetes Mellitus. Mediators Inflamm. 2010, 792393–792415. 10.1155/2010/792393 20634940 PMC2903979

[B117] Visser J. van Staden P. J. Soma P. Buys A. V. Pretorius E. (2017). The Stabilizing Effect of an Oligomeric Proanthocyanidin on Red Blood Cell Membrane Structure of Poorly Controlled Type II Diabetes. Nutr. Diabetes 7, e275. 10.1038/nutd.2017.25 28504711 PMC5518807

[B118] Wang L. Yamashita Y. Komeda S. Saito A. Ashida H. (2018). Absorption, Metabolism, Distribution and Faecal Excretion of B-type Procyanidin Oligomers in Mice after a Single Oral Administration of Black Soybean Seed Coat Extract. Food Funct. 9, 5362–5370. 10.1039/C8FO00852C 30264089

[B119] Watała C. Gwoździński K. (1993). Effect of Aspirin on Conformation and Dynamics of Membrane Proteins in Platelets and Erythrocytes. Biochem. Pharmacol. 45, 1343–1349. 10.1016/0006-2952(93)90288-8 8385461

[B120] Woulfe D. S. (2010). Akt Signaling in Platelets and Thrombosis. Expert Rev. Hematol. 3, 81–91. 10.1586/ehm.09.75 20352060 PMC2844717

[B121] Xie P. Cui L. Shan Y. Kang W. Y. (2017). Antithrombotic Effect and Mechanism of Radix *Paeoniae Rubra* . Biomed. Res. Int. 2017, 9475074. 10.1155/2017/9475074 28299338 PMC5337344

[B122] Xu Z. Du P. Meiser P. Jacob C. (2012). Proanthocyanidins: Oligomeric Structures with Unique Biochemical Properties and Great Therapeutic Promise. Nat. Prod. Commun. 7, 381–388. 10.1177/1934578x1200700321 22545414

[B123] Yamamoto E. Nishimura N. Okada K. Sekido C. Yamamichi S. Hasumi K. (2011). Inhibitors of Autoactivation of Plasma Hyaluronan-Binding Protein (Factor VII Activating Protease). Biol. Pharm. Bull. 34, 462–470. 10.1248/bpb.34.462 21467630

[B124] Yang H. Xiao L. Yuan Y. Luo X. Jiang M. Ni J. (2014). Procyanidin B2 Inhibits NLRP3 Inflammasome Activation in Human Vascular Endothelial Cells. Biochem. Pharmacol. 92, 599–606. 10.1016/j.bcp.2014.10.001 25450671

[B125] Yang K. Zhang L. Liao P. Xiao Z. Zhang F. Sindaye D. (2020). Impact of Gallic Acid on Gut Health: Focus on the Gut Microbiome, Immune Response, and Mechanisms of Action. Front. Immunol. 11, 580208. 10.3389/fimmu.2020.580208 33042163 PMC7525003

[B126] Yisimayili Z. Guo X. Liu H. Xu Z. Abdulla R. Akber Aisa H. (2019). Metabolic Profiling Analysis of Corilagin *In Vivo* and *In Vitro* Using High-Performance Liquid Chromatography Quadrupole Time-Of-Flight Mass Spectrometry. J. Pharm. Biomed. Anal. 165, 251–260. 10.1016/j.jpba.2018.12.013 30562708

[B127] Yu X. Chu S. Hagerman A. E. Lorigan G. A. (2011). Probing the Interaction of Polyphenols with Lipid Bilayers by Solid-State NMR Spectroscopy. J. Agric. Food Chem. 59, 6783–6789. 10.1021/jf200200h 21574575 PMC3124816

[B128] Zakrzeska A. Gromotowicz-Popławska A. Szemraj J. Szoka P. Kisiel W. Purta T. (2015). Eplerenone Reduces Arterial Thrombosis in Diabetic Rats. J. Renin Angiotensin Aldosterone Syst. 16, 1085–1094. 10.1177/1470320313515037 25031293

[B129] Zhang C. (2008). The Role of Inflammatory Cytokines in Endothelial Dysfunction. Basic Res. Cardiol. 103, 398–406. 10.1007/s00395-008-0733-0 18600364 PMC2705866

[B130] Zhang L. Wang Y. Li D. Ho C. T. Li J. Wan X. (2016). The Absorption, Distribution, Metabolism and Excretion of Procyanidins. Food Funct. 7, 1273–1281. 10.1039/C5FO01244A 26814915

[B131] Zhang Y. DeWitt D. L. Murugesan S. Nair M. G. (2004). Novel Lipid-Peroxidation- and Cyclooxygenase-Inhibitory Tannins from *Picrorhiza Kurroa* Seeds. Chem. Biodivers. 1, 426–441. 10.1002/cbdv.200490036 17191857

[B132] Zhang Y. Shi H. Wang W. Ke Z. Xu P. Zhong Z. (2011). Antithrombotic Effect of Grape Seed Proanthocyanidins Extract in a Rat Model of Deep Vein Thrombosis. J. Vasc. Surg. 53, 743–753. 10.1016/j.jvs.2010.09.017 21095090

[B133] Zheng B. Chen D. Yang X. Igo L. P. Li Z. Ye X. (2016). Development and Validation of an UPLC-PDA Method for the Determination of Corilagin in Rat Plasma and its Application to Pharmacokinetic Study. J. Chromatogr. B Analyt Technol. Biomed. Life Sci. 1031, 76–79. 10.1016/j.jchromb.2016.07.039 27459126

[B134] Zou M.-H. Cohen R. A. Ullrich V. (2004). Peroxynitrite and Vascular Endothelial Dysfunction in Diabetes Mellitus. Endothelium 11, 89–97. 10.1080/10623320490482619 15370068

